# A Differentiated SH-SY5Y Model of Hypoxic–Ischaemic Injury Reveals Dynamic Transcriptomic Responses During Reoxygenation

**DOI:** 10.3390/pathophysiology33030043

**Published:** 2026-06-25

**Authors:** Maryam Adenike Salaudeen, Stuart M. Allan, Emmanuel Pinteaux

**Affiliations:** 1 Division of Neuroscience, Faculty of Biology, Medicine, and Health, School of Biological Sciences, University of Manchester, Manchester M13 9PL, UK; maryam.salaudeen@manchester.ac.uk (M.A.S.); stuart.allan@manchester.ac.uk (S.M.A.); 2 Geoffrey Jefferson Brain Research Centre, Manchester Academic Health Science Centre, Northern Care Alliance NHS Foundation Trust, University of Manchester, Manchester M13 9PL, UK; 3 Department of Pharmacology and Therapeutics, Ahmadu Bello University, Zaria 810211, Nigeria

**Keywords:** neuronal injury, neuronal model, differentiated SH-SY5Y cells, hypoxia, ischaemia, hypoxic–ischaemic injury, oxygen-glucose deprivation

## Abstract

Background: Hypoxic–ischaemic brain injury (HI) is a major contributor to neurological deficits following stroke. Understanding what happens to the smallest functional and structural unit of the central nervous system in the face of oxygen and nutrient deprivation is essential to fully comprehend the pathogenesis of diseases and disorders associated with HI, such as ischaemic stroke. Aim: The aim of this study was to develop a robust in vitro tool for initial screening of potential therapeutics and identification of diagnostic markers of brain hypoxic injury. Methods: This study details and validates a comprehensive protocol for modelling HI using differentiated SH-SY5Y neuroblastoma cells (Neuron-like Cells, NLCs). First, we optimized the differentiation process and confirmed the maturity and purity of NLCs via standard molecular markers. The NLCs exhibited functional excitotoxicity, demonstrating a graded cell death response to N-methyl-D-aspartate (NMDA), thus validating their functional application. To simulate HI, we initially optimized the oxygen-glucose deprivation (OGD) treatment using graded concentrations of CoCl_2_ (0.125 mM to 2 mM) in glucose-free media. The validated NLCs were then subjected to the refined OGD protocol (1 mM CoCl_2_ in glucose-free media) for 3 h, followed by various periods of reoxygenation (1 h, 3 h, 6 h, 12 h, 18 h, and 24 h). Result: Bulk RNA-sequencing revealed a distinct temporal transcriptional response to HI. Injury-associated genes, including heat shock proteins and stress markers, were significantly (*p* < 0.05) upregulated at 3 h of reoxygenation, peaked at 6 h, and declined thereafter, remaining above baseline at 24 h. Upstream regulator analysis identified IL-1β, TNF-α, and HIF-1α as key drivers during OGD, with additional regulators emerging during reoxygenation. TNF-α and β-oestradiol were consistently identified across time points, while TGF-β1 and NTRK1 became prominent during peak injury and later phases. Analysis of secreted factors showed increased release of inflammatory (TNF-α) and neurotrophic (β-NGF, BDNF, VEGF) mediators with reoxygenation, while maximal cell death occurred at 24 h. Conclusions: This study identifies a transient, time-dependent transcriptional cascade following hypoxic–ischaemic injury, highlighting a critical window for early neuronal response. The model provides a reproducible platform for studying neuronal injury and recovery, and identifies known (TNF-α, IL-β, and HIF-1α), context-specific (NTRK1 and TGF-β) and novel (β-oestradiol) regulators of the injury response with potential relevance for therapeutic targeting.

## 1. Introduction

**Figure 1:**
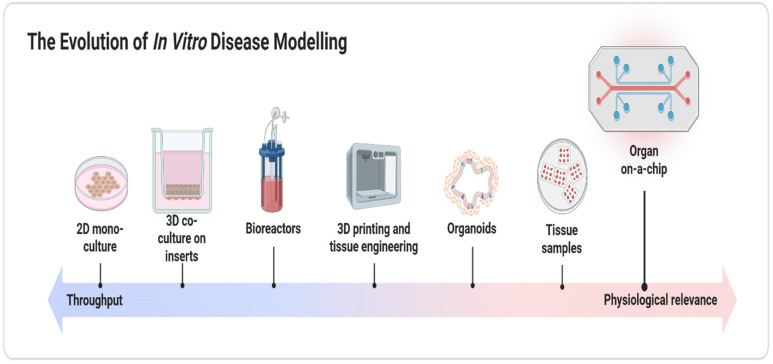
The evolution of in vitro disease models. Various in vitro disease models exist, and with increasing understanding and technological advancement, these models have evolved from simple 2D monolayer cultures to highly complex and sophisticated organ-on-chips. Each generation of model offers peculiar advantages and limitations, and all have been invaluable to understanding the pathophysiology of diseases, drug discovery, and disease diagnosis. Image was created in Biorender. Salaudeen, M. (2026) https://BioRender.com/1yghy4i, accessed on 15 April 2026.

For decades, in vitro disease models have laid the foundation for unravelling the complexities of neurological disorders, offering critical insights into cellular mechanisms contributing to their aetiology, progression, and potential treatments. However, the complex nature of these diseases demands increasingly robust preclinical models to accurately capture the nuances of central nervous system (CNS) damage. In response, in vitro techniques have evolved over time—from simple 2D monolayer cultures, cocultures, organoids and more recently organ-on-a-chip [1,2,3,4] (Figure 1). With this progress, these models are increasingly relevant to humans, enabling a better understanding of disease mechanisms. Paradoxically, advancements often come with escalating costs and complexity [5,6,7], creating significant barriers to access, particularly for research institutions in emerging economies [8,9]. This disparity inhibits global research potential and can lead to inefficient resource allocation in the broader neuroscience community.

Hypoxic–ischaemic brain injury (HI) remains a major cause of mortality and long-term neurological disability following events such as cardiac arrest, hypoxic-ischaemic encephalopathy, stroke, and traumatic brain injury [10,11,12,13]. Despite significant advances in critical care, effective neuroprotective therapies remain limited, largely due to the complex and dynamic cellular responses triggered during oxygen deprivation and subsequent reperfusion. Importantly, neuronal injury is not driven solely by the initial hypoxic insult but also by the cascade of inflammatory, oxidative, and reparative processes that occur during reperfusion and reoxygenation [14]. Consequently, understanding the temporal dynamics of these responses requires experimental models capable of recapitulating both injury and recovery phases.

Neuroscience research relies heavily on the use of immortalized cell lines derived from both rodents and humans. Human NT2 and SH-SY5Y cells, as well as rat PC12 cells, are some of the most widely used neuronal cell lines. The neuroblastoma SH-SY5Y cells are more commonly employed for brain-related studies because of their robustness, scalability, human origin, and capacity for neuronal differentiation [15,16,17]. Several laboratories have modelled hypoxic-ischaemic injury (HI) using SH-SY5Y cells. These studies, however, mostly utilize undifferentiated SH-SY5Y cells or are silent about the differentiation state of the cells [18,19,20]. A recent review by Voogd et al. highlighted this issue, reporting that more than 90% of in vitro studies using this cell line rely on undifferentiated cultures [21]. While these models have provided valuable insights, undifferentiated SH-SY5Y cells retain proliferative characteristics that may not fully reflect the biology of mature neurons. In contrast, differentiated SH-SY5Y cells exhibit several characteristics of mature post-mitotic neurons and may, therefore, offer improved physiological relevance when investigating neuronal responses to hypoxic-ischaemic injury. Accordingly, this study utilizes differentiated SH-SY5Y cells as a practical compromise between biological relevance, experimental reproducibility, and accessibility. Unlike primary neuronal cultures, which are often very expensive and technically demanding to establish and maintain, differentiated SH-SY5Y cells provide a robust and versatile platform supported by well-established differentiation protocols.

A major hurdle in modelling hypoxic and ischaemic insults in vitro is achieving stable, reproducible oxygen deprivation [22,23]. Although physical oxygen-glucose deprivation systems remain the gold standard for modelling hypoxia, their implementation often requires specialized infrastructure, including hypoxia chambers or dedicated hypoxia workstations, and can be expensive and difficult to integrate with high-throughput workflows [23,24,25,26,27]. These practical limitations can restrict the detailed investigation of cellular events which often precede gene-level changes.

To address these challenges, cobalt chloride (CoCl_2_) has emerged as a widely used chemical hypoxia mimetic capable of inducing hypoxia-responsive pathways through inhibition of prolyl hydroxylase domain enzymes and subsequent stabilisation of hypoxia-inducible factor-1α (HIF-1α) [28]. While CoCl_2_ has been extensively used to model hypoxic responses in a variety of cellular systems, its application in differentiated SH-SY5Y cells remains comparatively underexplored [27,29,30]. In the present study, we establish and characterize a reproducible CoCl_2_-based model of hypoxic–ischaemic injury in differentiated neuron-like SH-SY5Y cells and investigate the associated transcriptomic responses across multiple reoxygenation time points (1, 3, 6, 12, 18, and 24 h). By combining an accessible hypoxia-induction strategy with temporal transcriptomic profiling, this work provides a practical framework for investigating neuronal responses to hypoxic–ischaemic injury and recovery while maintaining experimental accessibility for laboratories with limited resources.

## 2. Methods

### 2.1. SH-SY5Y Cell Expansion

The neuroblastoma cell line SH-SY5Y cells (Merk Life Science, Cambridge, UK; RRID of CVCL_0019; Cat. # 94030304-1VL) were obtained at seventh passage (P7) from the manufacturer. The cells were subsequently seeded and expanded in T75 flasks until needed. Expansion was done in high-glucose Dulbecco’s Modified Eagle’s Medium (DMEM, Gibco, Loughborough, UK) supplemented with 10% foetal bovine serum (FBS, Gibco, Paisley, UK) and 1% streptomycin (1000 μg/mL)–penicillin (100 IU) (Sigma-Aldrich, Dorset, UK). This medium will be referred to as complete DMEM (cDMEM) from here on. Once about 80% confluent, the cells were passaged to maintain a healthy population.

### 2.2. SH-SY5Y Differentiation

The protocol by Dravid et al. (2024) [31] was adopted. Briefly, T75 flasks were coated with 10% Matrigel (Corning, Deeside, UK). The Matrigel-containing flasks were then incubated at 37 °C and 5% CO_2_ for 1 h. Thereafter, approximately 6 × 10^5^ SH-S5Y cells in cDMEM were seeded into a T75 flask and incubated at 37 °C and 5% CO_2_ for 24 h. Thereafter, the medium was replaced with stage I medium (refer to Table 1 for a full list of medium composition) and incubated at the same temperature and CO_2_ condition for 5 days. By the end of this incubation period, cells were visibly dispersed with emerging neurite outgrowth (Supplementary Figure S1). The stage I medium was then replaced with stage II medium (see Table 1) and incubated similarly for 5 days. By this time, the cells appeared fully differentiated and were maintained in stage II medium. Differentiated cells were subsequently referred to as neuron-like cells (NLCs). For all experiments, NLCs were used between passage 1 and 2 (P1–P2).

### 2.3. Assessment of NLC Maturity, Relative Purity, and Functional Integrity

#### 2.3.1. Neuronal Purity and Maturity

To determine the maturity of the differentiated NLCs, immunocytochemistry was performed to investigate the expression of markers of mature neurons such as β-III tubulin, microtubule-associated protein 2 (MAP2), synaptophysin (SYN), and neuronal nuclei (NeuN). The absence of glial fibrillary acidic protein (GFAP) was also confirmed to rule out astrocytic contamination of the cells.

#### 2.3.2. Functional Assay: Assessment of NLC Response to NMDA-Induced Excitotoxicity

NLCs at P0 to P1 were seeded in Matrigel-coated 96-well plates at a density of 3 × 10^4^ cells/well. Once the neurite outgrowth was well established, the cells were treated with graded concentrations (31.25 to 500 µM) of NMDA (Tocris, Bristol, UK) for 3 h in custom-made Magnesium-free media (145 mM NaCl, 5 mM KCl, 2 mM CaCl_2_, 20 mM HEPES, 5 mM Glucose, 10 µM Glycine. The pH was adjusted to 7.4, and the solution was sterile filtered using 0.22 µm syringe filter)). Thereafter, the supernatant was collected to measure cell death via membrane damage (using LDH assay), while metabolic activity was used to assess cell viability by treating the cells with 3-(4,5-dimethylthiazol-2-yl)-2,5-diphenyltetrazolium bromide solution (MTT). (See “LDH Assay” and “MTT Assay” for details). These experiments were repeated three (3) times on separate biological cultures.

### 2.4. Modelling Hypoxic–Ischaemic Injury on NLCs

#### 2.4.1. Phase I: Identifying Optimal Hypoxia-Inducing Concentration of Cobalt Chloride

NLCs were seeded in Matrigel-coated 24-well plates at a density of ~5.8 × 10^3^ cells/cm^2^. Once the neurite outgrowth was well established, the cells were treated with OGD media (see Table 1) with increasing concentrations (0.125 to 2 mM) of CoCl_2_. The cells were then incubated at 37 °C and 5% CO_2_ for 3 h. At the end of the incubation period, the medium was aspirated and replaced with a stage II medium for 24 h. Thereafter, supernatants were collected to assess cell death, inflammation, and hypoxia by measuring LDH, tumour necrosis factor alpha (TNF-α), chemokine ligand 2 (CCL2), and vascular endothelial growth factor (VEGF), respectively. This experiment was repeated three (3) more times on separate cultures.

#### 2.4.2. Phase II: Investigating Time-Course of Reoxygenation-Induced Neuronal Injury

Following result analysis, 1 mM cobalt chloride was selected as the optimal concentration for the induction of hypoxia in NLCs. Hence, NLCs at P1 to P2 were seeded at a density of ~8 × 10^3^ cells/cm^2^ or 3 × 10^4^ cells/well in Matrigel-coated 24-well and 96-well plates, respectively. The cells were treated with OGD medium containing 1 mM CoCl_2_ for 3 h at 37 °C and 5% CO_2._ This was then followed by replacement with reoxygenation media [oxygen-glucose deprivation and reoxygenation (OGD/R) media; see “Table 1”] for various times. Reoxygenation time points (1, 3, 6, 12, 18, and 24 h) were selected a priori to capture the immediate (1–3 h), intermediate (6–12 h), and late (18–24 h) phases of neuronal responses following OGD. This experimental design enabled the identification of both transient and sustained transcriptional changes occurring during the reoxygenation period.

At the end of reoxygenation, culture supernatants were collected and assayed for cell death (LDH and MTT), neuroinflammation (by measuring TNF-α and CCL2 secretion), and intrinsic hallmarks of neurogenesis (measuring brain-derived neurotrophic factor (BDNF), β-nerve growth factor (β-NGF), and VEGF). RNA was extracted from the cells and used for bulk RNA sequencing. The experiment was repeated four (4) more times, and RNA sequencing was done using RNA extracted from cells in the 2nd and 4th repeat experiments (randomly selected).

#### 2.4.3. Immunocytochemistry

For all immunocytochemistry, the NLCs were fixed sequentially in graded concentrations of paraformaldehyde (PFA)—first in 1% PFA for 5 min, then in 2% PFA for a similar time, and finally in 4% PFA for 10 min. Sequential PFA fixation was necessary to avoid disrupting the cytoskeleton and preserve the neurite morphology (as we have observed in our laboratory). Thereafter, the cells were washed briefly (~1 min) with phosphate-buffered saline (PBS) and then permeabilized with 0.2% PBS-Triton X (PBST, Sigma-Aldrich; Dorset, UK) for 10 min. This was followed by a 1 h block with 2% bovine serum albumin (BSA) (Sigma-Aldrich, Dorset, UK) in 0.2% PBST. At the end of the 1 h incubation, the cells were washed twice with wash buffer (0.1% PBS-Tween20) for a total of 5 min. The cells were then treated with primary antibodies (see “Table 2”) diluted in 2% BSA for 1 h. Thereafter, the wash cycle was repeated, and the cells were treated with secondary antibodies (see “Table 2”) and 0.1 µg/mL DAPI (1 in 20 dilution).

#### 2.4.4. Lactate Dehydrogenase Assay

The degree of neuronal death following different experiments was analysed using an LDH assay kit (Promega, Southampton, UK) according to the manufacturer’s instructions. Briefly, culture media supernatants from both treated and untreated cells were collected and mixed with assay buffer at room temperature for 30 min, protected from light. LDH was measured at a wavelength of 492 nm, and optical densities were normalized to a 100% cell death control. The measured LDH concentrations were compared with control values (untreated cells). The release of LDH is directly proportional to cell death.

#### 2.4.5. MTT Assay

The viability of NLCs following treatment with NMDA or exposure to oxygen–glucose deprivation and reoxygenation (OGD/R) was measured using the MTT assay. This involved adding 2.5 mg/mL of MTT solution to the cells and incubating in the dark at 37 °C and 5% CO_2_ for 3 h. This was followed by carefully removing the solution and then dissolving the purple formazan with 200 μL MTT solvent per well. The absorbance was then read at 590 nm, and optical densities were normalized to 100% viable control (untreated NLCs). Cell viability is directly proportional to the measured optical density.

#### 2.4.6. Enzyme-Linked Immunosorbent Assay

Levels of human BDNF, β-NGF, VEGF, TNF-α and CCL2 in NLC culture supernatants were quantified by ELISA using DuoSet^®^ kits (R&D Systems, Oxford, UK) according to the manufacturer’s instructions. Detection limits were 31.2 pg/mL for β-NGF, 15.6 pg/mL for TNF-α, CCL2, and VEGF, and 23.4 pg/mL for BDNF. For each assay, the samples were diluted as needed, and protein levels were calculated against a four-parameter logistic (4-PL) curve fit on CLARIOstar^®^ microplate reader software (430–2488; BMG LABTECH, Ortenberg, Germany).

#### 2.4.7. RNA Extraction

At the end of OGD/R, supernatants were collected while total RNA was extracted from the cells using a PureLink™ RNA extraction minikit (Invitrogen, Waltham, MA, USA) according to the manufacturer’s instructions. The RNA extracted from all experimental groups was quantified using a Nanodrop 1000 spectrophotometer (Thermo Scientific, Wilmington, DE, USA).

#### 2.4.8. RNA-seq Library Preparation and Sequencing

Total RNA from two independent experiments was submitted to the Genomic Technologies Core Facility (GTCF), University of Manchester. Although a limited number of biological replicates (*n* = 2 per group) were used, stringent quality control indicated high consistency between replicates, as evidenced by clustering in principal component analysis. The dataset was therefore considered suitable for exploratory transcriptomic analysis. Quality and integrity of the RNA samples were assessed using a 4200 TapeStation (Agilent Technologies, Manchester, UK), and libraries were then generated using the Illumina^®^ Stranded mRNA Prep. Ligation kit (Illumina, Inc., Cambridge, UK) according to the manufacturer’s protocol. Briefly, total RNA (typically 0.025–1 µg) was used as input material from which polyadenylated mRNA was purified using poly-T, oligo-attached, magnetic beads. Next, the mRNA was fragmented under elevated temperature and then reverse transcribed into first-strand cDNA using random hexamer primers and in the presence of Actinomycin D (thus improving strand specificity while mitigating spurious DNA-dependent synthesis). Following removal of the template RNA, second-strand cDNA was then synthesized to yield blunt-ended, double-stranded cDNA fragments. Strand specificity was maintained by the incorporation of deoxyuridine triphosphate (dUTP) in place of dTTP to quench the second strand during subsequent amplification. Following a single adenine (A) base addition, adapters with a corresponding, complementary thymine (T) overhang were ligated to the cDNA fragments. Pre-index anchors were then ligated to the ends of the double-stranded cDNA fragments to prepare them for dual indexing. A subsequent PCR amplification step was then used to add the index adapter sequences to create the final cDNA library. The adapter indices enabled the multiplexing of the libraries, which were pooled prior to loading onto the appropriate flow-cell. This was then paired-end sequenced (59 + 59 cycles, plus indices) on an Illumina NovaSeq6000 instrument (Cal, USA). Finally, the output data was demultiplexed and BCL-to-Fastq conversion performed using Illumina’s bcl2fastq software, version 2.20.0.422.

#### 2.4.9. RNA-seq Data Analysis

The stranded paired-end RNA-seq reads were quality assessed using FastQC (v0.12.1; [32]) and FastQ Screen (v0.15.3; [33]), followed by adapter and low-quality base trimming with BBDuk from the BBMap suite (v38.96; [34]). Trimmed reads were mapped against the human reference genome (GRCh38) and gene annotation from Gencode (v49) using STAR (v2.7.10a; [35]). The “--quantMode GeneCounts” option was used to obtain read counts per gene from STAR.

Differential gene expression analysis was performed using the Bioconductor package DESeq2 (v1.26.0; [36]) with alpha = 0.05. Additionally, the lfcShrink function (with apeglm method) was applied to generate more accurate log2 fold change estimates. Normalized counts were obtained using the counts function (with normalized = TRUE), where the raw counts were normalized using the median-of-ratios method as described in [37]. Data transformation was performed using the rlog function to obtain regularized logarithm-stabilizing transformation expression values. The transformed values were used to perform principal component analysis using the prcomp function from the built-in R package stats.

#### 2.4.10. Statistical Analysis

Statistical analysis and graph designs were performed using GraphPad Prism software version 9.3.1 for Windows (Cal, USA). Data distribution and homogeneity of variance were assessed using Shapiro–Wilk and Levene’s tests, respectively. Normally distributed data with homogeneous variance were analysed using parametric one-way ANOVA. Where there was a significant difference between mean values (*p* < 0.05), the Tukey post hoc test was performed to determine where the difference lay. All values are expressed as mean ± standard error of the mean (SEM). With the exception of the RNA sequencing and unless stated otherwise, all experiments were performed on at least four independent culture/biological replicates, derived from separate SH-SY5Y differentiation experiments. Measurements obtained from the same differentiation batch were treated as technical replicates and averaged prior to statistical analysis.

#### 2.4.11. Image Analysis

Fluorescence images were analysed using Fiji (Version 1.5r; National Institutes of Health, Bethesda, MD, USA). For all analyses, identical image acquisition settings and image processing parameters were applied across experimental groups. NeuN-positive cells were quantified from thresholded NeuN fluorescence images using the Analyse Particles function in Fiji. The number of NeuN-positive objects was normalized to the total number of DAPI-stained nuclei within the corresponding field of view and expressed as a percentage of total nuclei.

Neurite length was quantified from βIII-tubulin-stained images. Following background correction and thresholding, images were converted to binary masks and skeletonized using the Skeletonize plugin in Fiji. Total neurite length was determined using the Analyse Skeleton plugin and normalized to the total number of DAPI-positive nuclei within the same field of view to account for differences in cell density between experimental groups. To assess overall neuronal network coverage, βIII-tubulin-positive area fraction was determined from thresholded βIII-tubulin images using the Measure function in Fiji/ImageJ (Version 1.54r) and expressed as the percentage of the image area occupied by βIII-tubulin-positive staining.

Quantification was performed on images obtained from at least three independent biological replicates per experimental condition.

## 3. Results

### 3.1. Differentiated SH-SY5Y Cells Have Neuron-like Characteristics and Express Functional NMDA Receptors

**Figure 2:**
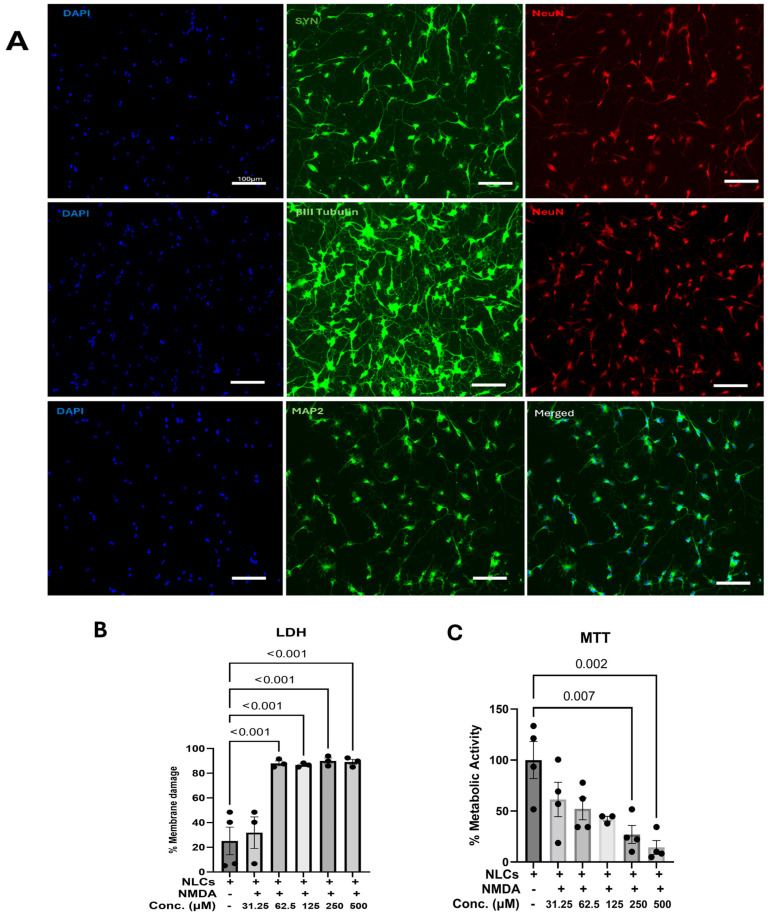
Differentiated SH-SY5Y cells Express Markers of Matured Neurons and were Susceptible to NMDA-induced Excitotoxicity. SH-SY5Y cells were differentiated into NLCs using RA and BDNF over a 10-day period. The differentiated cells stained positively for markers of mature neurons such as βIII tubulin, NeuN, SYN, and MAP2 (**A**). The NLCs also displayed a concentration-dependent degree of cell viability and cell death (**B**–**C**) when treated with graded concentrations of NMDA for 3 h. Images were captured using a Nikon Eclipse high-end widefield microscope at ×20 magnification using DAPI, FITC, and Texas Red filters. Scale Bar: 50 µm. NMDA data were analysed using one-way ANOVA followed by Tukey’s post hoc test. Results are presented as Mean ± S.E.M. Key: NLCs = Neuron-like cells; NeuN = Neuronal nuclei, SYN = Synaptophysin, MAP2 = Microtubule-associated protein 2, NMDA = N-methyl-D-Aspartate, RA = trans-Retinoic acid, BDNF = Brain-derived neurotrophic factor, ANOVA = Analysis of variance, S.E.M. = Standard error of mean, N = 4 experimental repeats.

Differentiated NLCs expressed various markers of neuronal maturity—NeuN, SYN, MAP2, and βIII tubulin—and did not stain positive for the GFAP antibody (Figure 2A). Treatment of these cells with increasing concentrations of NMDA resulted in a statistically significant (*p* < 0.05), concentration-dependent increase in membrane damage (Figure 2B), alongside decreasing metabolic activity (Figure 2C). At the lowest concentration tested (31.25 µM), NMDA exposure induced detectable membrane damage, as evidenced by increased LDH release, while approximately 50% of metabolic activity was retained relative to untreated controls. Metabolic activity continued to decline with increasing concentrations of NMDA, with only <15% of NLCs surviving the 500 µM NMDA after a 3 h treatment. (Figure 2C).

### 3.2. Exposure of NLCs to Glucose Deprivation in the Presence of 1 mM CoCl_2_ Caused Significant Hypoxic Injury

**Figure 3:**
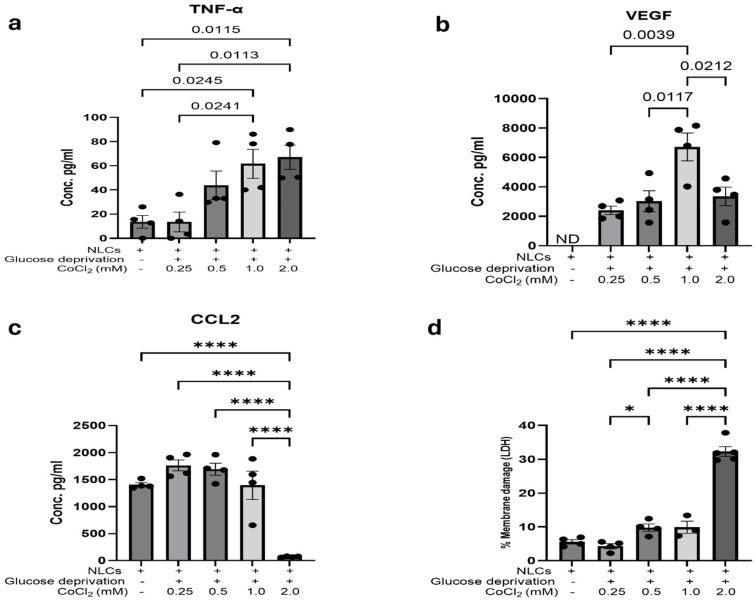
1 mM Cobalt chloride in glucose-free neurobasal media induced significant neuroinflammation with moderate cell death. Differentiated SH-SY5Y cells were treated with antibiotics and KCl-supplemented glucose-free Neurobasal media in the presence of graded concentrations of cobalt chloride for 3 h to simulate OGD. Thereafter, the supernatants were assayed for inflammation (by measuring TNF-α and CCL2), hypoxia (via VEGF secretion), and cell death. Compared to the untreated control, NLCs exposed to OGD produced a significant amount of TNF-α (**a**) and VEGF (**b**) with increasing concentrations of CoCl_2_ in the media. The secretion of CCL2 was only significantly affected when the concentration of CoCl_2_ in the media increased to 2 mM (**c**). Neuronal death was comparable to that in the untreated control when CoCl_2_ was <0.5 mM but increased significantly at a 2 mM concentration of CoCl_2_ in the media (**d**). Data were analysed using one-way ANOVA followed by Tukey’s post hoc test. Results are presented as Mean ± S.E.M. * = *p* < 0.05; **** = *p* < 0.0001. Key: KCl = Potassium chloride, OGD = Oxygen–glucose deprivation, TNF-α = Tumour necrosis factor-alpha, CCL2 = Chemokine ligand 2, VEGF = Vascular endothelial growth factor, NLCs = Neuron-like cells, CoCl_2_ = Cobalt chloride, ANOVA = Analysis of variance, S.E.M = Standard error of mean, N = 4 experimental repeats.

Exposing NLCs to OGD for 3 h using increasing concentrations of CoCl_2_ in glucose-free Neurobasal-A media resulted in the release of TNF-α and VEGF. Compared to untreated cells and cells deprived only of glucose, a 1 mM concentration of CoCl_2_ caused a significant (*p* < 0.05) ~3-fold increase in the secretion of TNF-α (Figure 3a). Similarly, CoCl_2_ stimulated significant VEGF synthesis and release into the culture supernatant compared to untreated cells, with maximal effect obtained with 1 mM CoCl_2_ (Figure 3b). CCL2 (a major neuronal chemokine) was highly expressed in untreated cells; however, only the highest concentration of CoCl_2_ (2 mM) significantly (*p* < 0.05) suppressed its secretion (Figure 3c), which correlated with significant membrane damage and potential cytotoxicity at this concentration only (Figure 3d).

### 3.3. Chemical Hypoxia Accelerates Neuronal Death and Interferes with the Functional and Structural Integrity of Neuron-like Cells

**Figure 4:**
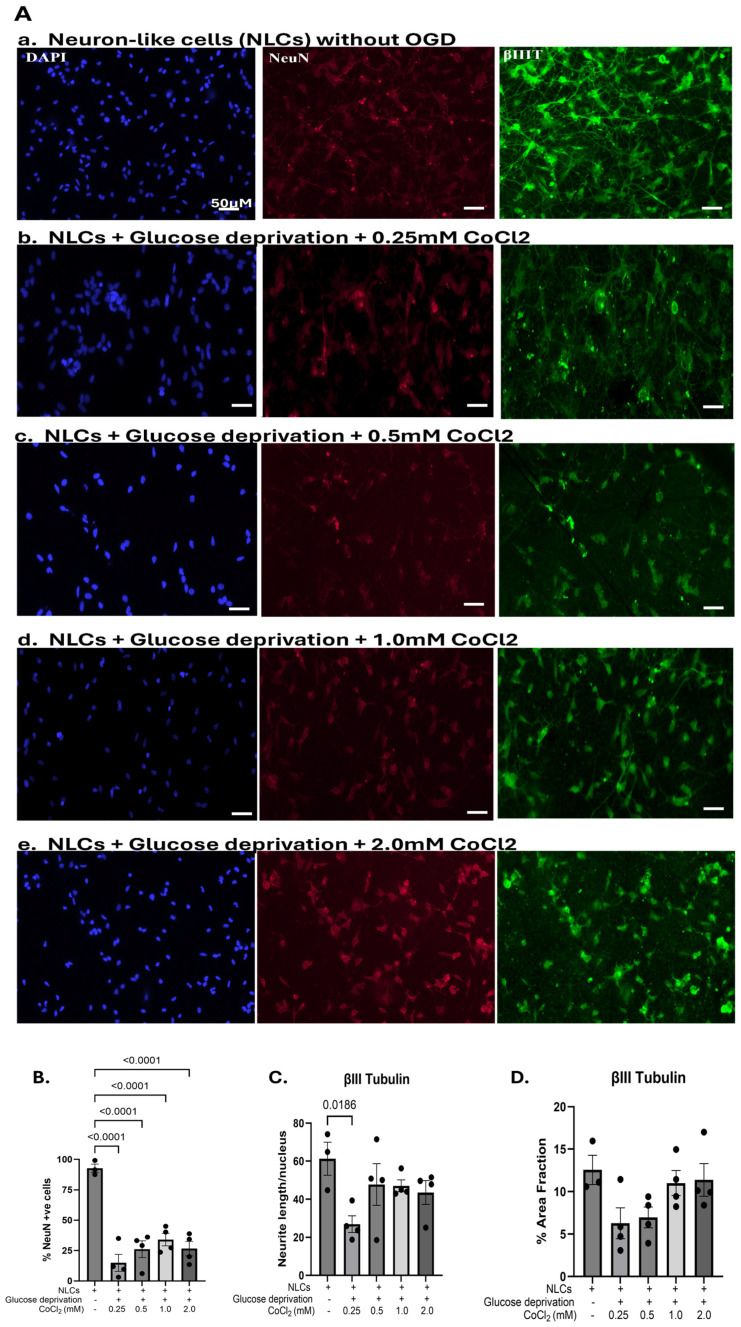
Effect of OGD modelled by CoCl_2_ on the expression of mature neuronal markers. (**A**) Representative fluorescence micrographs of differentiated neuron-like cells (NLCs) exposed to increasing concentrations of CoCl_2_ (0.25–2.0 mM). Nuclei were stained with DAPI (blue), the mature neuronal marker NeuN was stained red, and the neuronal cytoskeletal marker βIII-tubulin was stained green. (**B**) Quantification of NeuN-positive cells. (**C**) Quantification of neurite length normalized to total nuclei number. (**D**) Quantification of βIII-tubulin-positive area fraction. Data are presented as mean ± SEM with individual biological replicates shown. Statistical analyses were performed using one-way ANOVA followed by Dunnett’s multiple comparisons test. Scale Bar: 50 µm. Images were captured using Nikon Eclipse high-end widefield microscope. Key: SEM = Standard error of mean; ANOVA = Analysis of variance.

Depriving NLCs of glucose and oxygen resulted in significant cell death that was worsened in a concentration-dependent manner (Figure 4). Notably, compared to untreated NLCs, which expressed a high density of healthy, uniform-shaped viable cells (Figure 4A, panel a), NLCs that were exposed to glucose deprivation (GD) and oxygen deprivation exhibited clear changes in nuclei number and morphology. At all tested CoCl_2_ concentrations, OGD resulted in a visible decrease in NeuN staining intensity (Figure 4A, panels b–e) with a corresponding significant decrease in percentage NeuN +ve cell counts (Figure 4B). The lowest concentration of CoCl_2_ resulted in the most profound decrease in nuclei number that appeared condensed, with a corresponding decrease in NeuN staining intensity (Figure 4A, panel b) and NeuN-positive cells (Figure 4B). In parallel, βIII tubulin also appeared to be less dense with lower staining intensity compared to untreated NLCs. As the severity of hypoxic injury increased, cell density appeared to decrease. Increasing CoCl_2_ from 0.5 mM to 2 mM (Figure 4A, panels c–e) resulted in a diminished nuclear staining intensity. Whilst the lowest concentration of CoCl_2_ resulted in the least neurite length per nucleus (Figure 4C), increasing the concentration generally led to a comparable increase in neurite length per nucleus within the hypoxic groups (Figure 4C) and an increased percentage area fraction of βIII tubulin (Figure 4D).

Although neurite length and βIII-tubulin area fraction analyses revealed significant alterations at the lowest CoCl_2_ concentration, concentration selection was not based on a single morphological endpoint. Rather, the final concentration was chosen following consideration of the overall biological response, including cell viability, morphological integrity, and the ability to generate a robust yet non-lethal injury state suitable for downstream transcriptomic characterization. Based on these criteria, 1 mM CoCl_2_ provided the most appropriate balance between injury induction and preservation of sufficient cellular integrity for subsequent analyses.

### 3.4. Reoxygenation Following 3 h OGD Resulted in a Temporal Change in Proinflammatory Chemokine and Angiogenesis Factor, VEGF

**Figure 5:**
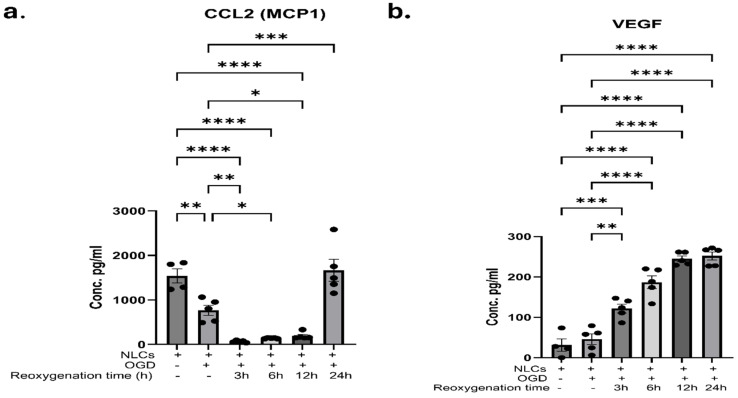
Translational effect of OGD on CCL2 and VEGF was most prominent at the 24 h reoxygenation period. Compared to NLCs that were exposed to OGD only, the reoxygenation of NLCs from 3 h to 12 h after OGD caused a remarkable (*p* < 0.05) drop in CCL2 production. This trend was completely and significantly (*p* < 0.05) reversed when reoxygenation extended to 24 h (**a**). On the other hand, reoxygenation resulted in a significant (*p* < 0.05) and time-dependent increase in the secretion of VEGF (**b**). Data were analysed using a one-way ANOVA followed by Tukey’s post hoc test. Results are presented as Mean ± S.E.M. * = *p* < 0.05; ** = *p* < 0.01, *** = *p* <0.001, **** = *p* <0.0001. Key: OGD = Oxygen–glucose deprivation, CCL2 = Chemokine ligand 2, VEGF = Vascular endothelial growth factor, NLCs = Neuron-like cells, ANOVA = Analysis of variance, S.E.M. = Standard error of mean, *N* = 5 experimental repeats.

Compared to the untreated NLCs, CCL2 secretion reduced significantly during OGD and increased gradually during reoxygenation, peaking at a level similar to that of the untreated cells after 24 h of reoxygenation. This level was statistically significantly higher (*p <* 0.05) than the OGD levels (Figure 5a). Similarly, 3 h of OGD resulted in a significant drop in VEGF levels compared to the untreated control, followed by a steady and significant rise with reoxygenation time (Figure 5b). Compared to the secretion of VEGF by NLCs exposed to OGD without reoxygenation, reoxygenation for 3, 6, 12, and 24 h resulted in ~2.7-fold, 4-fold, 5-fold, and 5.5-fold increase in VEGF secretion, respectively (Figure 5b). However, reoxygenation did not seem to have any major influence on the release of TNF-α (Supplementary Figure S2a), BDNF (Supplementary Figure S2b), and β-NGF (Supplementary Figure S2c) as their levels were comparable across all reoxygenation time points.

### 3.5. NLCs Undergo Sequential, Multi-Pathway Response Following a 24 h Time-Course OGD/R

**Figure 6:**
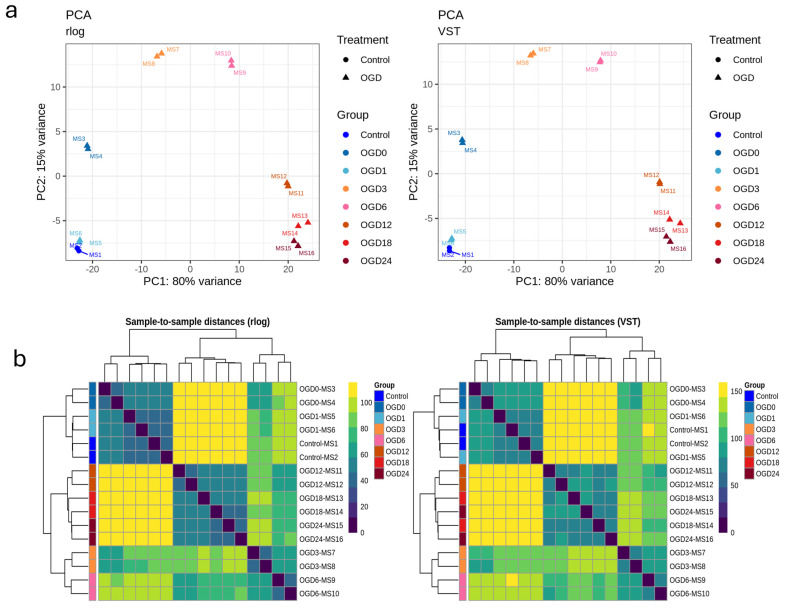
Comparative analysis of transcriptional activity and cellular dynamics in NLCs following OGD/R with a focus on the canonical pathways. PCA plots (rlog and VST) (**a**) showing clear separation between control and OGD samples and tight clustering of biological replicates. Sample-to-sample distance heatmaps (rlog and VST) (**b**) showing clustering of replicates and distinct separation between experimental conditions, consistent with PCA. Key: OGD = Oxygen–glucose deprivation, VST = variance-stabilizing transformation, PCA = Principal component analysis.

Principal component analysis (PCA) (Figure 6a) and sample-to-sample distance heatmaps (Figure 6b) of the RNA-seq data using both rlog and variance stabilising transformation (VST) revealed clear separation between control and OGD-treated samples along PC1, which accounted for 80% of the variance. Replicates clustered closely together, indicating good consistency between randomly selected biological samples. The consistency of clustering observed across both transformation methods supports the robustness of the dataset. Furthermore, OGD samples exhibited structured separation consistent with treatment duration, suggesting progressive transcriptional changes in response to hypoxic–ischaemic conditions. Similar clustering patterns were observed when PCA was performed on differentially expressed genes (Supplementary Figure S3).

Ingenuity pathway analysis (IPA) of the RNAseq data using the log2 fold change values of the gene counts revealed several upstream regulators (URs) in NLCs with changing activation states (Figure 6). Acute OGD triggered the initial activation of three primary signalling cascades: HIF-1α, Zn homeostasis, and cellular senescence. While a brief 1 h reoxygenation period appeared to suppress these stress signals, extended reoxygenation (6 h) induced a pronounced second wave of injury-associated signalling. This phase was characterized by the reactivation of the initial OGD-responsive pathways alongside the recruitment of diverse programmed cell death subroutines, including necroptosis, ferroptosis, and AMPK-mediated metabolic stress, as well as neuroinflammation. Notably, a transient activation of CREB signalling was observed at this stage, suggesting an early but futile survival response. However, by the late reoxygenation phase (12–24 h), this pro-survival CREB signalling was profoundly downregulated. This loss of survival signalling coincided with the terminal upregulation of pro-death execution pathways (pyroptosis and death receptor signalling), which occurred in parallel with a late-stage induction of compensatory repair mechanisms (TGF-β and Neurotrophin/TRK signalling).

**Figure 7:**
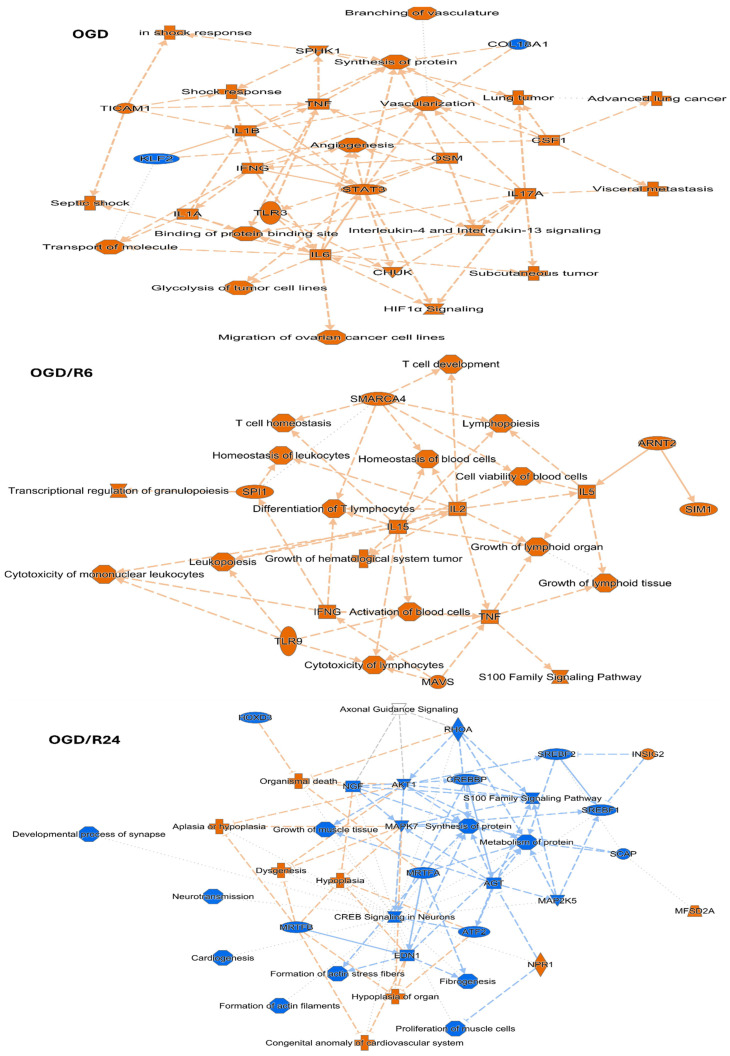
Network analysis reveals a transition from acute inflammatory signalling to late-stage regenerative programs in OGD/R-treated neurons. IPA-generated networks illustrate the functional connectivity between differentially expressed genes (DEGs) and biological pathways during OGD and reoxygenation at 6 h and 24 h. The initial OGD phase is dominated by pro-inflammatory and hypoxia-responsive hubs, including TNF, IL1B, and HIF-1α, which shift toward a transient transcriptional repression at 1 h of reoxygenation. Reoxygenation at 6 h is characterized by a surge in immune-like survival signalling, featuring activation of STAT-mediated pathways and Interleukin (IL2, IL15) hubs. The late-stage recovery is marked by a dual signature: the initiation of neuroregenerative programs, such as axonal guidance and synaptogenesis, which paradoxically coincides with the profound inhibition of the CREB Signalling master switch by 24 h. Key: Nodes represent genes or biological functions, with lines indicating documented biological interactions. Node and edge colours represent the predicted activation state based on experimental log2 fold change: orange indicates predicted activation (z-score > 0), while blue indicates predicted inhibition (z-score < 0). HIF = Hypoxia-inducible factor; TNF-α = Tumour necrotic factor alpha; IL-1B = Interleukin 1 beta; OGD = Oxygen-glucose deprivation; OGD/R = OGD and reoxygenation.

Furthermore, network analysis revealed a highly coordinated, time-dependent transition between distinct functional modules (Figure 7). During the initial OGD phase, the network was characterized by a robust activation of pro-inflammatory signalling hubs, including TNF, IL1β, and IL6. These nodes were central to pathways involving an endotoxin-like shock response and hypoxia-induced angiogenesis, indicating a rapid alarm response intrinsic to the NLCs. Immediately following the initiation of reoxygenation (1 h), a dramatic shift toward network-wide inhibition was observed (indicated by blue nodes). Key regulators such as EGF and TGF-β1 were predicted to be inhibited, suggesting a transient transcriptional stun or a protective metabolic pause as the cells adapted to the sudden influx of oxygen and associated oxidative stress. Between 3 h and 6 h of reoxygenation, the networks returned to an activated state (orange), dominated by pathways traditionally associated with immune cell maturation and lymphopoiesis. Despite the 2D monoculture environment, there was significant enrichment in STAT-mediated signalling and interleukin-related hubs (IL2, IL15, INFG, and TNF). This phase may reflect activation of neuronal-intrinsic survival pathways and increased expression of genes associated with neurotrophic signalling. As reoxygenation progressed into the late stages (12 h–24 h), the networks transitioned from survival signalling to structural and functional recovery. At 12 h, we observed the emergence of the CREB Signalling in Neurons module, a critical regulator of neuronal survival and plasticity. By 24 h, the network topology was dominated by nodes associated with axonal guidance signalling and synaptogenesis. Detailed hourly transcriptional changes are contained in Supplementary Figure S4.

**Figure 8:**
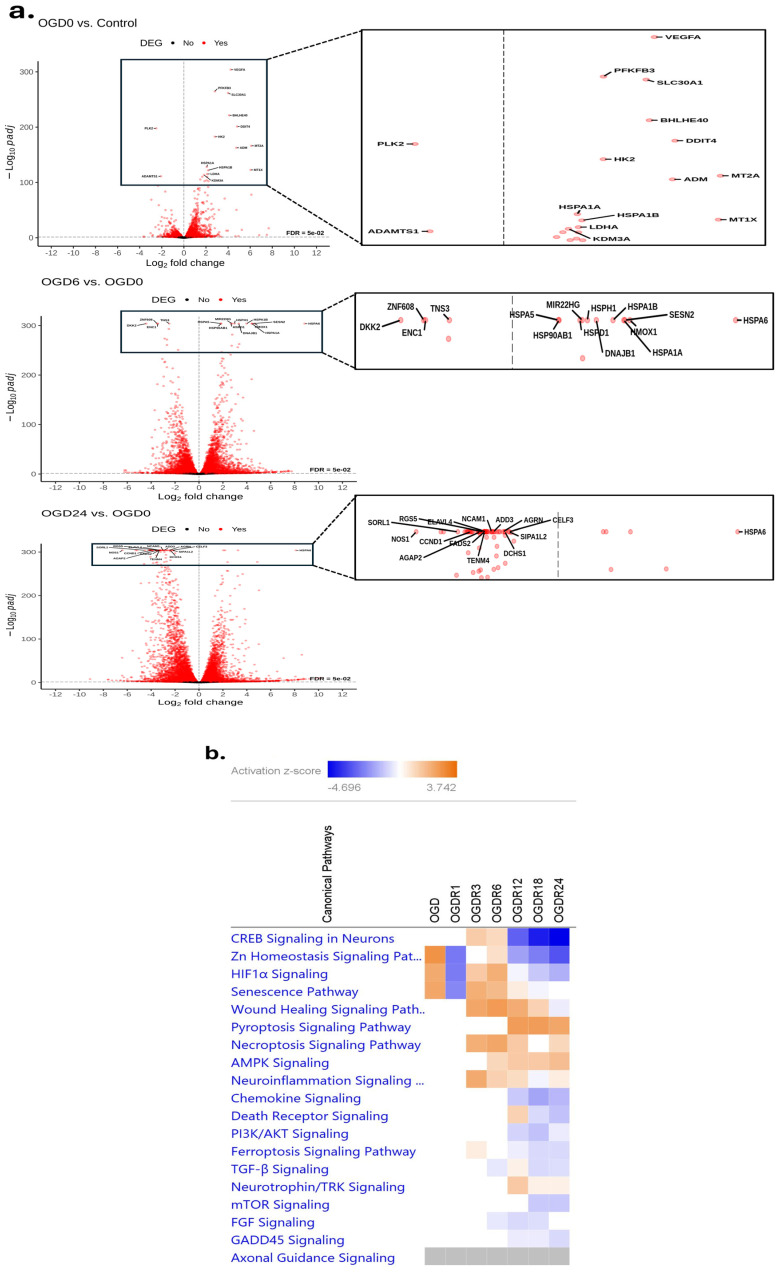
Dynamic transcriptional profiling of NLCs during OGD and progressive reoxygenation. Volcano plots illustrate the distribution of DEGs across all experimental conditions. The *x*-axis represents the log2 fold change (LFC), and the *y*-axis represents the statistical significance (log10 adjusted *p*-value). Red points indicate genes meeting the significance threshold ($FDR < 0.05). (**a**) Comparison of OGD vs. Control baseline. (b–g) Comparison of reoxygenation time points (6 h, and 24 h) relative to the OGD0 state. Representative top-regulated genes, including hypoxia-responsive elements (VEGFA), heat shock proteins (HSPA6), and neuronal markers (NOS1), are annotated. The heatmap (**b**) reveals clear variation in the activation of some stroke-relevant canonical pathways. OGD alone resulted in the activation of pathways involved in Zn homeostasis, senescence, and HIF-1α. Main reoxygenation injury started from 3 h of reoxygenation via the activation of various signalling pathways, including neuroinflammation, necroptosis, and wound healing. As reoxygenation progressed, more deleterious pathways (death receptor, pyroptosis) were activated with corresponding deactivation of beneficial signals such as CREB signalling in neurons, TGF-β, NTRK, and wound healing signals. Key: DEGs = differentially expressed genes, OGD = Oxygen-glucose deprivation, VEGFA = Vascular endothelial growth factor A, HSPA6 = Heat shock protein family A member 6; NOS = Nitric oxide synthase 1, CREB = cAMP response element-binding protein, TGF-β = Transforming growth factor beta, NTRK = Neurotrophic receptor tyrosine kinase.

To identify specific gene expression changes driving the observed pathway alterations, we performed differential expression analysis across the experimental timeline (Figure 8). Volcano plots revealed a triphasic expression pattern of the top 15 genes in each group when compared to the unstressed cells. This response was typically characterized by an initial downregulation, followed by a peak in expression and a subsequent return to near-baseline levels. Initial exposure to OGD (OGD0 vs. Control) resulted in significant upregulation of classic hypoxia-responsive genes, including VEGFA, PFKFB3, and SLC20A1, as well as cellular stress markers like DDIT4 and members of the Metallothionein family (MT1X, MT2A). (Figure 8a). Notably, at 1 h of reoxygenation (OGD1 vs. OGD0), we observed a rapid downregulation of the hypoxia-inducible gene signature, particularly VEGFA, reflecting the cellular response to oxygen restoration. As reoxygenation progressed into the intermediate phase (3 h–6 h), the transcriptional profile shifted toward a massive proteotoxic stress response. This was characterized by the high-magnitude upregulation of heat shock proteins, most notably HSPA6, which remained the most significantly upregulated gene through 24 h. By the late phase (12 h–24 h), the volcano plots revealed an increasing number of significantly downregulated genes (e.g., NOS1, RGS5, and CCND1), which correlates with the observed inhibition of synaptic and proliferative pathways such as CREB and PI3K/AKT signalling. This late-stage transcriptional divergence highlights a failure to restore homeostatic gene expression, favouring instead a profile associated with prolonged cellular stress and suppressed neuronal signalling. A volcano plot showing differentially expressed genes at each time point is contained in (Supplementary Figure S5).

## 4. Discussion

The present study introduces a standardized and cost-effective protocol for simulating hypoxic–ischaemic damage in differentiated human SH-SY5Y neuroblastoma cells. By leveraging the chemical hypoxia-mimetic cobalt chloride, we addressed the common hurdles of reproducibility and high costs associated with conventional hypoxia chambers. Our findings demonstrate that differentiated SH-SY5Y cells, referred to here as neuron-like cells (NLCs), provide a more biologically relevant and susceptible model for studying the mechanisms of mature post-mitotic neuronal injury compared to undifferentiated cells.

A critical aspect of our model is the successful differentiation of SH-SY5Y cells into mature NLCs. The expression of neuronal markers, including β-III tubulin, MAP2, SYN, and NeuN, confirms the neuronal maturity of these cells [38]. The absence of the glial marker GFAP is suggestive of minimal astrocytic contamination, although additional lineage markers would be required to definitively exclude progenitor or glial populations. The functional integrity of these NLCs was further validated through their concentration-dependent response to NMDA-induced excitotoxicity, a hallmark of neuronal injury [39,40]. This differentiation is essential, as mature neurons are the primary targets of excitotoxic and apoptotic damage during hypoxic–ischaemic events [41,42].

Our results identify 1 mM CoCl_2_ as the optimal concentration for inducing significant hypoxic stress without immediate massive cell death, allowing for the study of temporal cellular changes. CoCl_2_ effectively mimics hypoxia by inhibiting prolyl hydroxylase domain enzymes (PHDs), which prevents the degradation of hypoxia-inducible factor-1α (HIF-1α). This was corroborated by our observation of increased VEGF secretion, a downstream target of HIF-1α, in cells treated with CoCl_2_ during glucose deprivation [30,43,44]. Notably, TNF-α, BDNF, and β-NGF did not exhibit significant temporal changes following OGD/R despite showing modest fluctuations over the reoxygenation period. The absence of robust TNF-α responses may reflect the simplified neuronal monoculture employed in this study, as astrocytes and microglia are recognized as major contributors to inflammatory cytokine production following hypoxic–ischaemic injury [45,46]. Similarly, while BDNF and β-NGF play important roles in neuronal survival, plasticity, and repair, their expression may occur over longer recovery periods or depend on intercellular signalling mechanisms that are not fully represented in neuron-only cultures. In contrast, VEGF and CCL2 demonstrated pronounced temporal regulation, consistent with their established roles as early hypoxia-responsive mediators associated with HIF-dependent signalling and cellular stress responses [47,48]. These findings suggest that the early reoxygenation phase in differentiated SH-SY5Y cells is characterized predominantly by hypoxia-responsive and chemotactic signalling rather than robust inflammatory or neurotrophic factor induction.

Our whole-transcriptome analysis at 0 h (OGD without reoxygenation) revealed the simultaneous activation of HIF-1α, TNF-α, and IL-1β. While this observation is often seen in immune cells like microglia, studies have shown that neurons also undergo similar changes during ischaemic injury [49]. This precisely models the severe, acute phase of cerebral ischaemia, where oxygen deprivation (HIF-1α activation) immediately triggers a maximal, destructive neuroinflammatory response (TNF-α and IL-1β activation) [44,50]. This acute phase is likely responsible for the significant cell death observed in our assays. The concurrent activation of the Bmal1:clock pathway at this time suggests that the ischaemic insult immediately disrupts fundamental cellular circadian programs, adding another layer of metabolic vulnerability. Bmal1 is a natural clock known to protect against focal ischaemia via various mechanisms, including the reduction of oxidative stress and regulation of metabolism [51,52]. Its early emergence in our model identifies a potential ‘chronotherapeutic’ window, where the timing of intervention could be as critical as the treatment itself.

Shortly after reoxygenation (3 h), the acute inflammatory driver, IL-1β, was inhibited, while TNF-α and the potent systemic hormone, β-oestradiol, were reactivated. β-oestradiol is known for its neuroprotective and anti-inflammatory properties following stroke, suggesting the NLCs initiate a powerful endogenous mechanism to limit secondary injury [53,54]. The subsequent phase (6 h) sees the emergence of activated TGF-β and NTRK1 as main upstream regulators, followed by the activation of CREB signalling and axonal guidance signalling in the late reoxygenation phase (12–24 h). A notable finding here is the significant downregulation of genes traditionally associated with synaptic plasticity and vascular integrity, such as NCAM1, ELAVL4, and RGS5. While this phase saw the activation of CREB and axonal guidance pathways, the simultaneous suppression of these structural and synaptic genes may reflect a ‘metabolic conservation’ strategy. Following the exhaustion of the heat shock response (HSPs) by the 12th hour, the neurons may undergo a period of ‘synaptic stripping’ or functional dormancy to prioritize basic survival over energy-intensive dendritic remodelling. This suggests a critical window where the cell is transitioning between attempted repair and potential delayed degeneration. NTRK1 (the TrkA receptor) is a core component of neurotrophic support and survival. The most intriguing finding is the activation of TGF-β1 at the 6 h mark, a regulator universally recognized as the central mediator of fibrosis and glial scarring by activating astrocytes in vivo [55,56,57]. Interestingly, TGF-β1 was identified as a predicted upstream regulator despite the use of a predominantly neuronal monoculture. Given the well-established role of TGF-β signalling in tissue remodelling and repair, this observation may indicate the activation of intrinsic neuronal stress-response or structural adaptation programmes following hypoxic–ischaemic injury. One possible explanation is that differentiated SH-SY5Y cells retain the capacity to engage signalling networks that overlap with pathways traditionally associated with tissue repair and remodelling [58,59]. However, the precise biological significance of this response remains unclear and cannot be determined from transcriptomic data alone. Furthermore, whether similar responses occur in primary neurons or other neuronal model systems remains to be established. As such, these findings should be considered hypothesis-generating and warrant further mechanistic investigation. Nevertheless, the identification of TGF-β1-associated signalling highlights the utility of this differentiated SH-SY5Y model for uncovering candidate regulatory pathways involved in neuronal responses to HI and recovery.

Lastly, this model offers a robust and economical platform for neuroscientists, particularly in resource-limited settings, to investigate the cellular and molecular consequences of HI. While we acknowledge that a 2D monoculture cannot fully replicate the complex environment of the human brain, especially with respect to the interaction between neurons and glial cells, it serves as an invaluable and accessible entry point for mechanistic studies and high-throughput screening.

While this study provides important insights into the neuronal response to HI, some limitations should be considered when interpreting the findings. First, the use of a 2D monoculture system does not fully recapitulate the cellular complexity of the in vivo brain environment, particularly the interactions between neurons, astrocytes, microglia, and vascular components that are known to play critical roles in hypoxic–ischaemic injury. As such, the model primarily captures neuron-intrinsic responses and may not reflect the full spectrum of neuroinflammatory and repair processes observed in vivo.

Second, although CoCl_2_ is widely used as a hypoxia-mimetic agent, it does not fully replicate the physiological dynamics of oxygen deprivation and may introduce off-target effects independent of hypoxia signalling. Therefore, caution should be exercised when extrapolating these findings to physiological or clinical contexts.

Third, the RNA sequencing analysis was performed using a limited number of biological replicates (n = 2 per group), which may reduce statistical power and increase the risk of false positives. However, stringent quality control measures, including consistent clustering in PCA and sample-to-sample distance analyses, support the reproducibility of the dataset. Nonetheless, validation using independent experimental approaches remains essential.

Finally, while this study provides a detailed temporal transcriptional profile of neuronal responses to HI, the functional roles of the identified upstream regulators and signalling pathways were not directly interrogated. Future studies incorporating mechanistic validation, alternative neuronal model systems, and more complex model systems will be necessary to determine the extent to which these findings are conserved across different cellular contexts and to further elucidate the biological significance of the identified pathways.

## 5. Conclusions and Future Directions

Our robust, cost-effective model successfully mapped the time-dependent transcriptional consequences of HI. The identified temporal regulatory cascade highlights candidate pathways that may warrant further investigation as potential therapeutic targets. We recommend that future work focus on validating the protein expression of these key regulators (especially TGF-β1) and using this accessible platform to screen small molecules capable of disrupting the transition to the potentially detrimental “scarring” phase.

## Figures and Tables

**Table 1:** Various culture media for the differentiation of SH-SY5Y cells and induction of hypoxic–ischaemic injury in neuron-like cells.

Type of Media	Composition	Note
**Stage I**	For every 500 mL of high-glucose DMEM, add 12.5 mL heat-inactivated foetal bovine serum (FBS, Gibco) 5 mL penicillin/streptomycin (Gibco, Catalogue #15646-055) 10 µM trans-retinoic acid (RA) (Sigma, Darmstadt, Germany. Catalogue #R2625) 5 mL GlutaMAX^®^ (Gibco, ThermoFisher, Paisley, UK. Catalogue #35050-51)	Always add the RA fresh (just before use). Because of its poor aqueous solubility, prepare stocks of 2 mM in DMSO (Sigma) and use in the absence of light (turn off the hood light at the time of use).
**Stage II**	500 mL Neurobasal-A medium (Gibco, ThermoFisher Scientific, Loughborough, UK, 10888-022) 50 ng/mL recombinant human BDNF (Novus Bio, Oxford, UK, Cat. #248-BD or Proteintech, Manchester, UK, Cat. #HZ-1335) 20 mM KCL B-27 Supplement (1×) (Gibco, Paisley, UK, Cat. #17564-044) 5 mL GlutaMAX^®^ 5 mL penicillin/streptomycin	
**OGD Media**	500 mL glucose-free Neurobasal-A medium (Gibco, Cat. #A24775-01) 20 mM KCL (Fisher Chemicals; Illkirch Cedex, France) 5 mL GlutaMAX^®^ 5 mL penicillin/streptomycin Cobalt chloride (25 mM stock; Life Science; Saint-Quentin-Fallavier, France)	BDNF and B-27 were not included in the media.
**OGD/R Media**	500 mL Neurobasal-A medium (Gibco 10888-022) 20 mM KCL 5 mL GlutaMAX^®^ 5 mL penicillin/streptomycin	BDNF and B-27 were not included in the media.

**Table 2:** List of various primary and secondary antibodies used to assess the maturity and purity of neuron-like cells.

	Antibodies	Dilutions	Hosts	Manufacturer
Primary Antibodies	β III Tubulin	1:100	Mouse	Abcam (Ab78078); Cambridge, UK
	Neuronal Nuclei (NeuN)	1:100	Rabbit	Proteintech (26975-1-AP); Manchester, UK
	Growth-associated protein 43 (GAP43)	1:500	Chicken	Invitrogen (PA5-95660)
	Synaptophysin (SYN)	1:150	Mouse	Abcam (Ab8049); Cambridge, UK
	Microtubule-associated protein 2 (MAP2)	1:100	Goat	Abcam (Ab11267); Cam-bridge, UK
	Glial fibrillary acidic protein (GFAP)_	1:1000	Chicken	Antibodies (A85307)); Cambridge, UK
Secondary Antibodies	Alexa 647	1:2000	Chicken	Life technologies (A21449); Paisley, UK
	Alexa 647		Rabbit	Invitrogen (A31573); Paisley, UK
	Alexa 488		Mouse	Invitrogen (A11001); Paisley, UK
	Alexa 488		Chicken	Invitrogen (A78948)
	Alexa 647		Anti-mouse	Invitrogen (A31571); Paisley, UK
	Alexa 647		Anti-goat	Invitrogen (A21447)

## Data Availability

RNA-seq data have been deposited in the ArrayExpress database at EMBL-EBI under accession number E-MTAB-16459 (https://www.ebi.ac.uk/biostudies/arrayexpress/studies/E-MTAB-16459, accessed on 18 December 2025). Raw data for all other experiments can be found in the University of Manchester repository, Figshare, via the https://doi.org/10.48420/30915899.

## Supplementary Materials

The following supporting information can be downloaded at https://www.mdpi.com/article/10.3390/pathophysiology33030043/s1, Figure S1: Principal component analysis (PCA) based on differentially expressed genes (DEGs); Figure S2: Translational Effect of OGD on TNF-α, BDNF, and β-NGF after various hours of Reoxygenation; Figure S3: Principal component analysis (PCA) based on differentially expressed genes (DEGs); Figure S4: Network Analysis Reveals a Transition from Acute Inflammatory Signalling to Late-stage Regenerative Programs in OGD/R-treated Neurons; Figure S5: Dynamic Transcriptional Profiling of NLCs During OGD and Progressive Reoxygenation.
