# Supplementary material for: A Differentiated SH-SY5Y Model of Hypoxic–Ischaemic Injury Reveals Dynamic Transcriptomic Responses During Reoxygenation
Source: Pathophysiology. 2026 Jun 25;33(3):43. doi: 10.3390/pathophysiology33030043 (PMC13397833; doi:10.3390/pathophysiology33030043)
Supplement: Supplementary file 1 [file pathophysiology-33-00043-s001.zip › pathophysiology-4292216-supplementary.pdf]

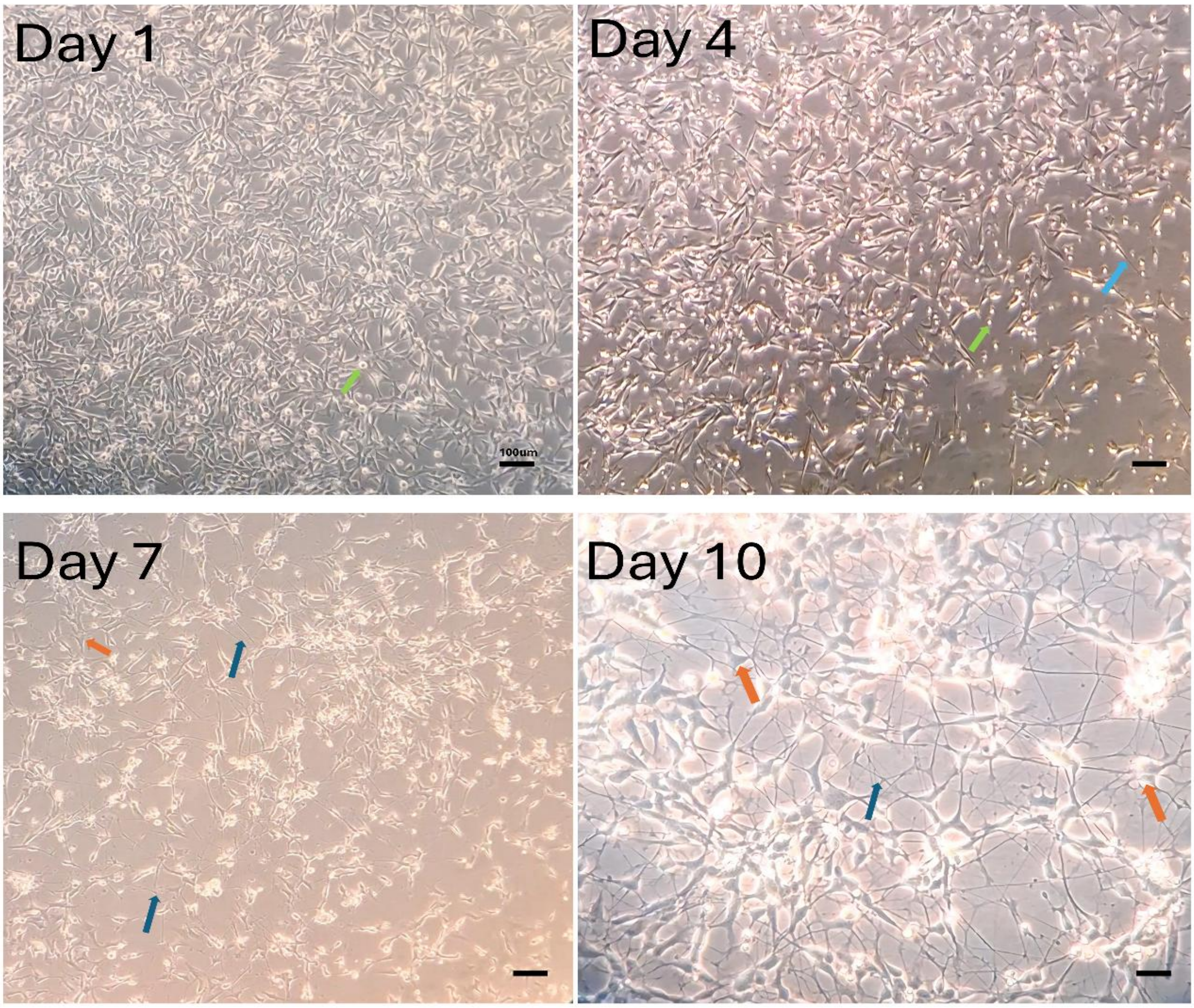

Supplementary Figure S1: Morphological Progression of SH-SY5Y Cell Differentiation into Neuron-like Cells (NLCs).

Representative images showing the temporal progression of SH-SY5Y differentiation. At Day 1, cells exhibit typical undifferentiated morphology. By Day 3, retinoic acid (RA) treatment induces the emergence of neurite-like projections. At Day 7, cells display pronounced neurite extension with visible intercellular connections. By Day 10, cells exhibit mature neuron-like morphology with extensive neurite networks and defined soma, indicating successful differentiation. Representative bright-field images were captured to illustrate morphological changes during differentiation

Key: Green arrow ↑= Epithelial cells, light blue arrow ↑ = emerging neurite; blue arrow↑ = Neurite extensions; Orange arrow ↑ = Cell body/soma

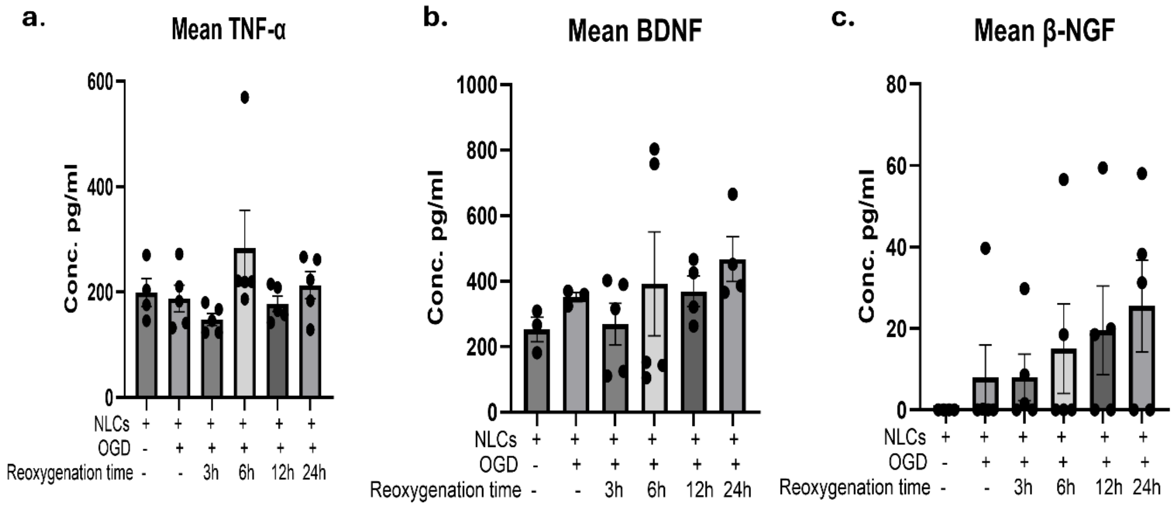

Supplementary Figure S2: Translational Effect of OGD on TNF-α, BDNF, and β-NGF After Various Hours of Reoxygenation

Compared to NLCs that were exposed to OGD only, the reoxygenation of NLCs from 3 h to 24 h after OGD did not affect the secretion of TNF-α (a). Although reoxygenation for 6 h resulted in much higher secretion of BDNF compared to the other time points, this rise was not statistically significant (b). Similarly, while not statistically significant, reoxygenation resulted in time-dependent increase in B-NGF release into the culture supernatant (c). Data were analysed using one-way ANOVA followed by Tukey post-hoc test. Results are presented as Mean ± S.E.M

Key: OGD = Oxygen-glucose deprivation, TNF-α = Tumour necrosis factor- alpha, BDNF = Brain-derived Neurotrophic factor, NGF = Nerve growth factor, NLCs = Neuron-like cells, $CoCl_2$ = Cobalt chloride. ANOVA = Analysis of variance, S.E.M = Standard error of mean, N = 5 experimental repeats.

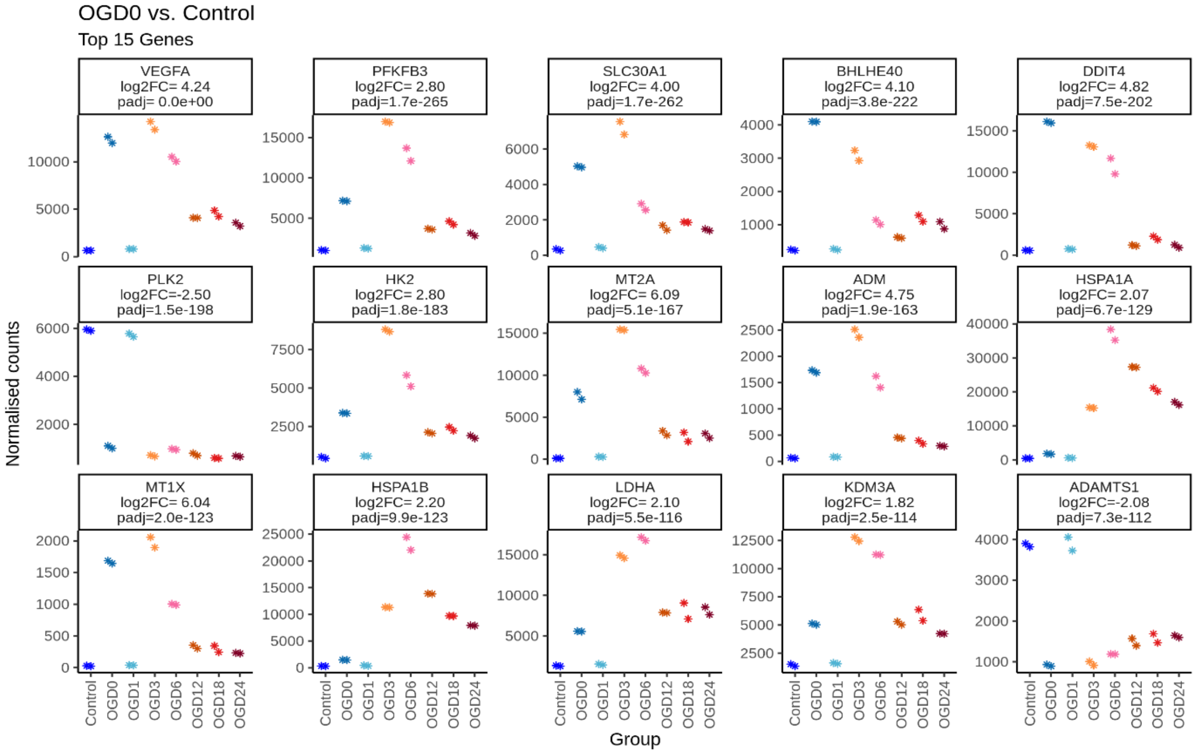

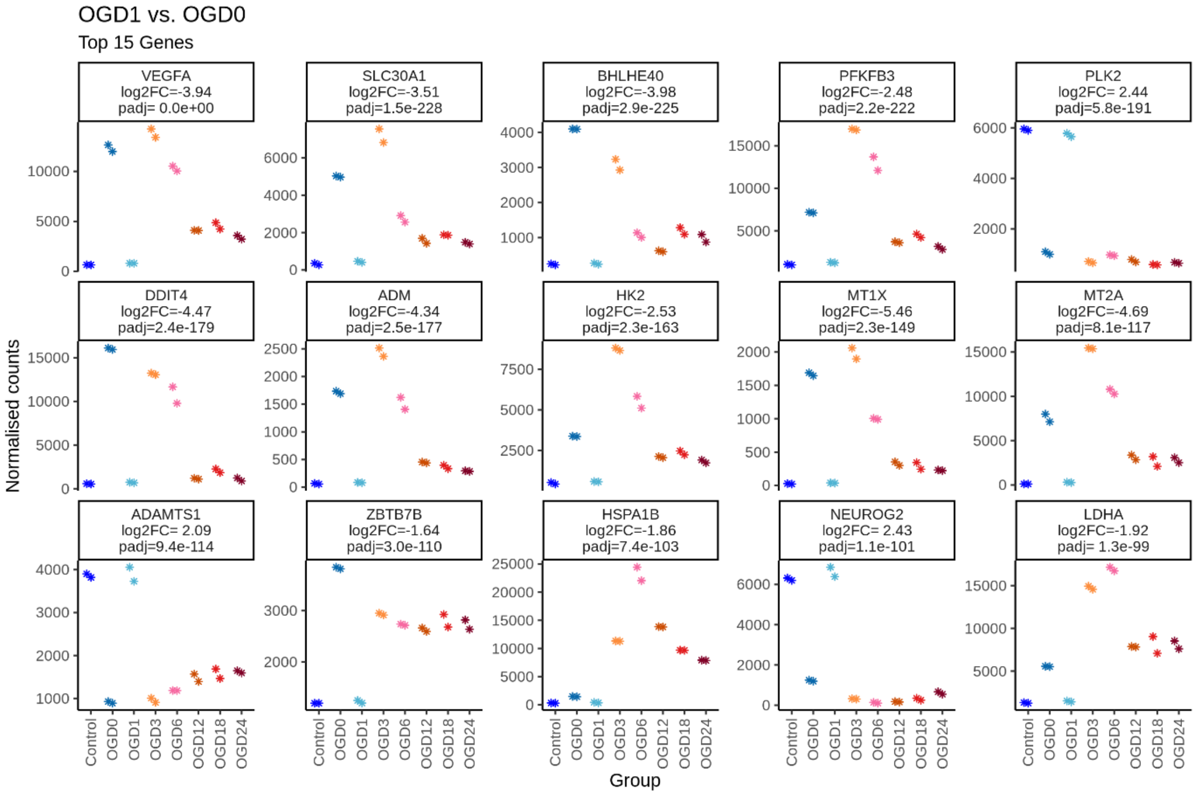

Figure continues to the next page

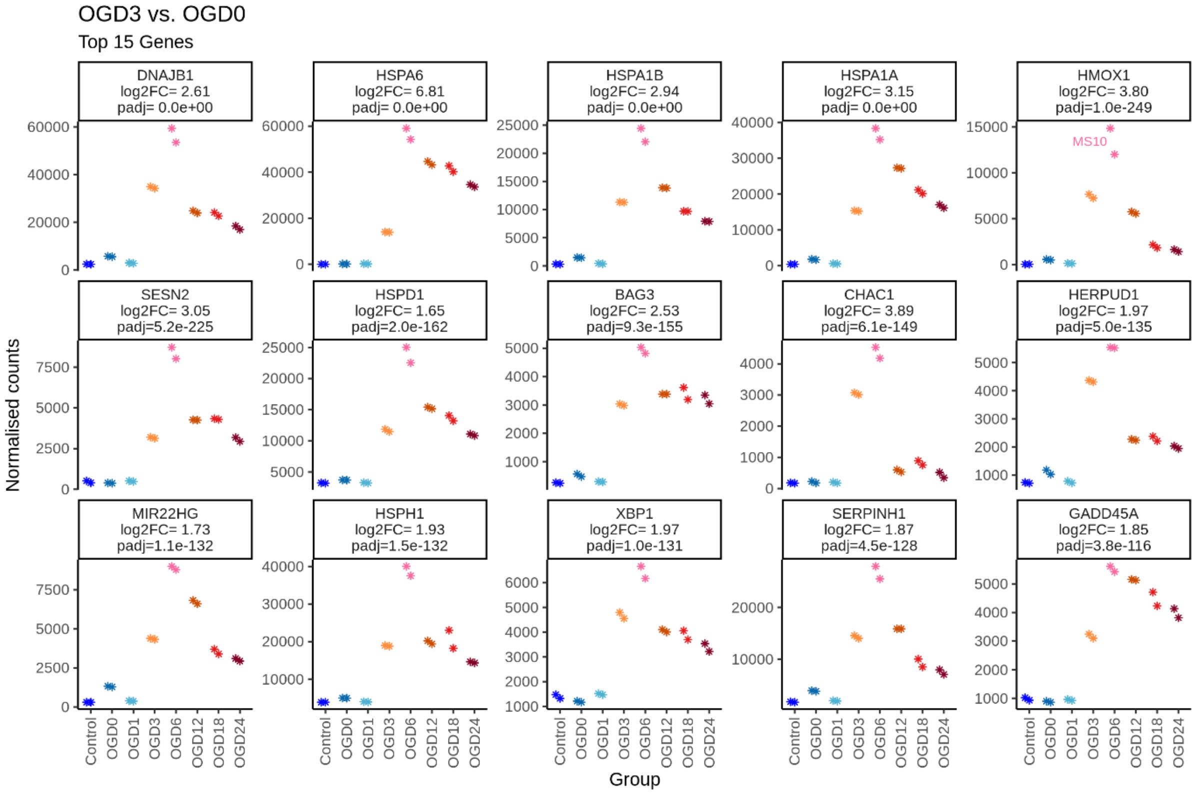

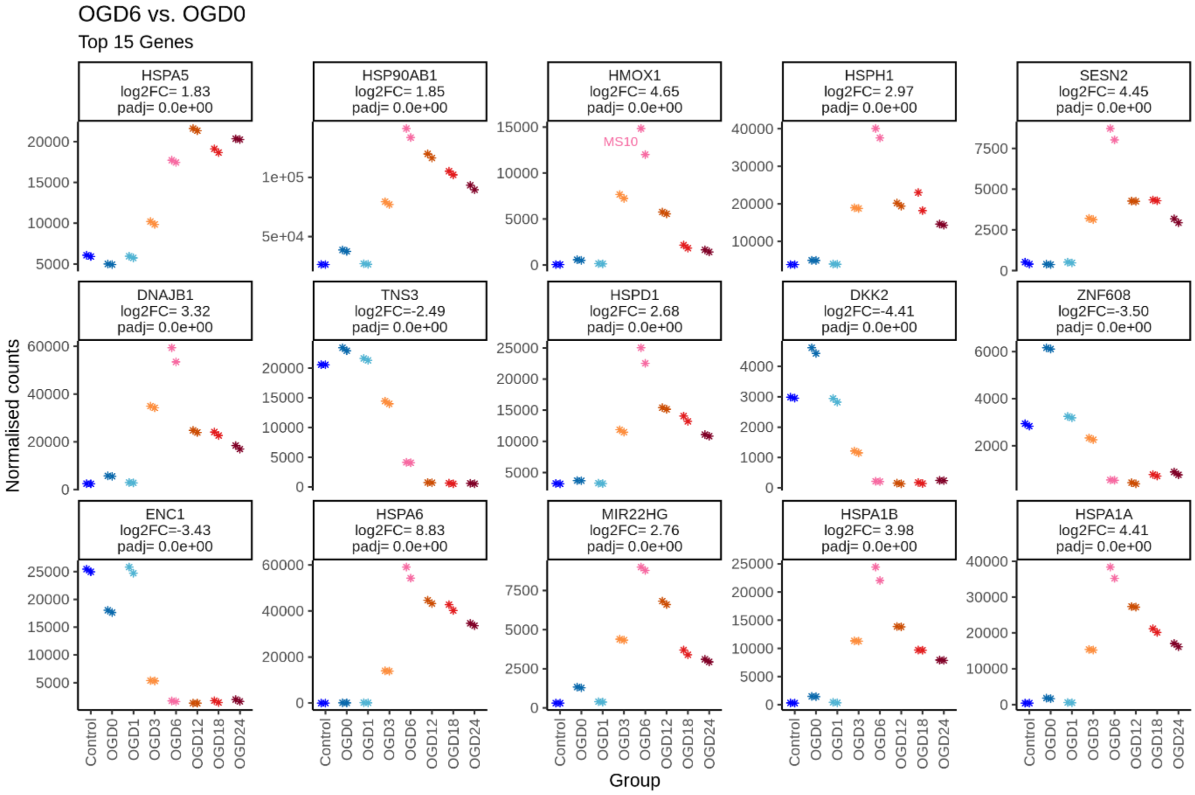

Figure continues to the next page

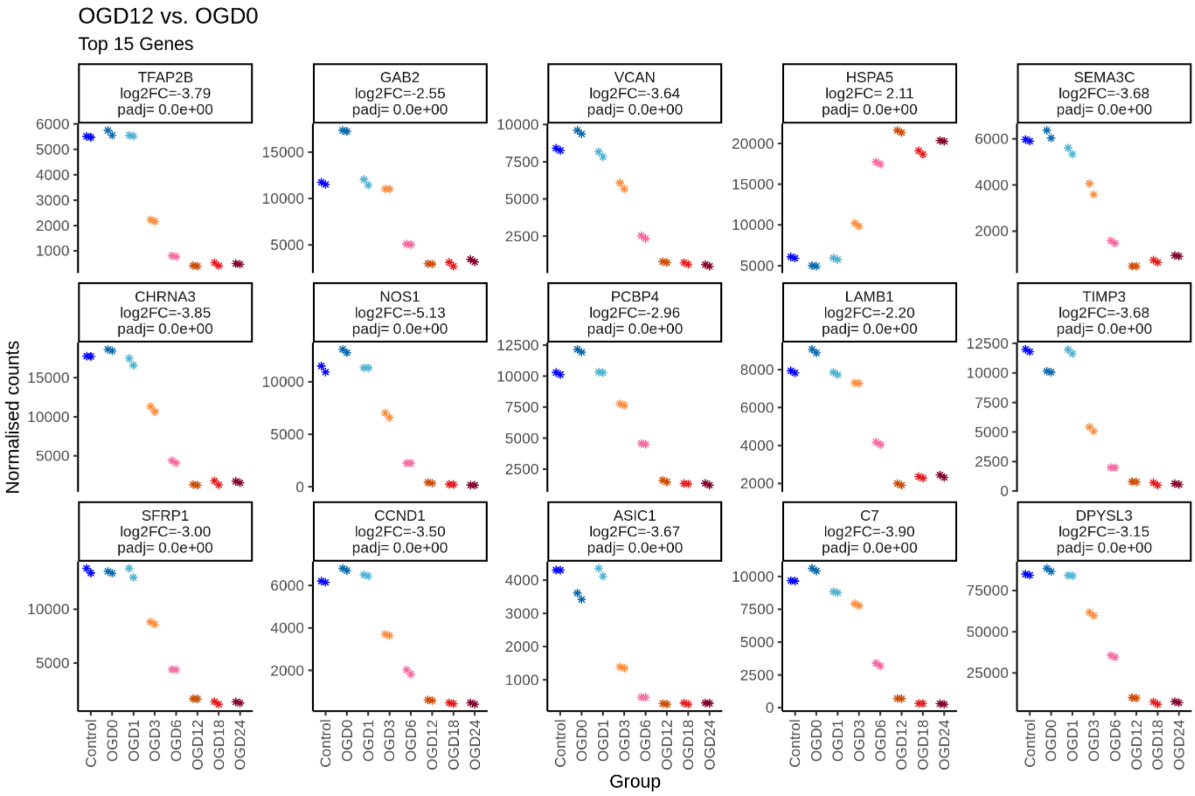
OGD12 vs. OGD0
Top 15 Genes
TFAP2B log2FC=-3.79 padj= 0.0e+00
GAB2 log2FC=-2.55 padj= 0.0e+00
VCAN log2FC=-3.64 padj= 0.0e+00
HSPA5 log2FC= 2.11 padj= 0.0e+00
SEMA3C log2FC=-3.68 padj= 0.0e+00
CHRNA3 log2FC=-3.85 padj= 0.0e+00
NOS1 log2FC=-5.13 padj= 0.0e+00
PCBP4 log2FC=-2.96 padj= 0.0e+00
LAMB1 log2FC=-2.20 padj= 0.0e+00
TIMP3 log2FC=-3.68 padj= 0.0e+00
SFRP1 log2FC=-3.00 padj= 0.0e+00
CCND1 log2FC=-3.50 padj= 0.0e+00
ASIC1 log2FC=-3.67 padj= 0.0e+00
C7 log2FC=-3.90 padj= 0.0e+00
DPYSL3 log2FC=-3.15 padj= 0.0e+00
Normalised counts
Control OGD0 OGD1 OGD3 OGD6 OGD12 OGD18 OGD24
Group

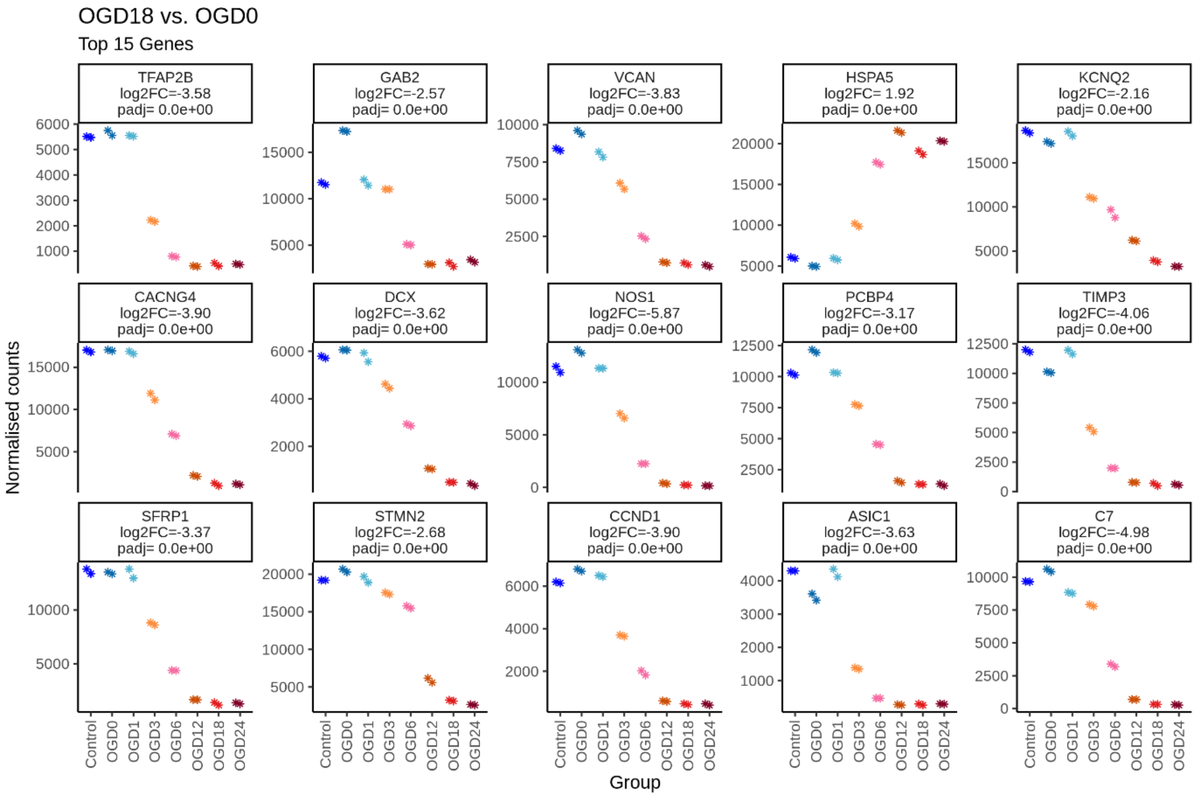
OGD18 vs. OGD0
Top 15 Genes
TFAP2B log2FC=-3.58 padj= 0.0e+00
GAB2 log2FC=-2.57 padj= 0.0e+00
VCAN log2FC=-3.83 padj= 0.0e+00
HSPA5 log2FC= 1.92 padj= 0.0e+00
KCNQ2 log2FC=-2.16 padj= 0.0e+00
CACNG4 log2FC=-3.90 padj= 0.0e+00
DCX log2FC=-3.62 padj= 0.0e+00
NOS1 log2FC=-5.87 padj= 0.0e+00
PCBP4 log2FC=-3.17 padj= 0.0e+00
TIMP3 log2FC=-4.06 padj= 0.0e+00
SFRP1 log2FC=-3.37 padj= 0.0e+00
STMN2 log2FC=-2.68 padj= 0.0e+00
CCND1 log2FC=-3.90 padj= 0.0e+00
ASIC1 log2FC=-3.63 padj= 0.0e+00
C7 log2FC=-4.98 padj= 0.0e+00
Normalised counts
Control OGD0 OGD1 OGD3 OGD6 OGD12 OGD18 OGD24
Group

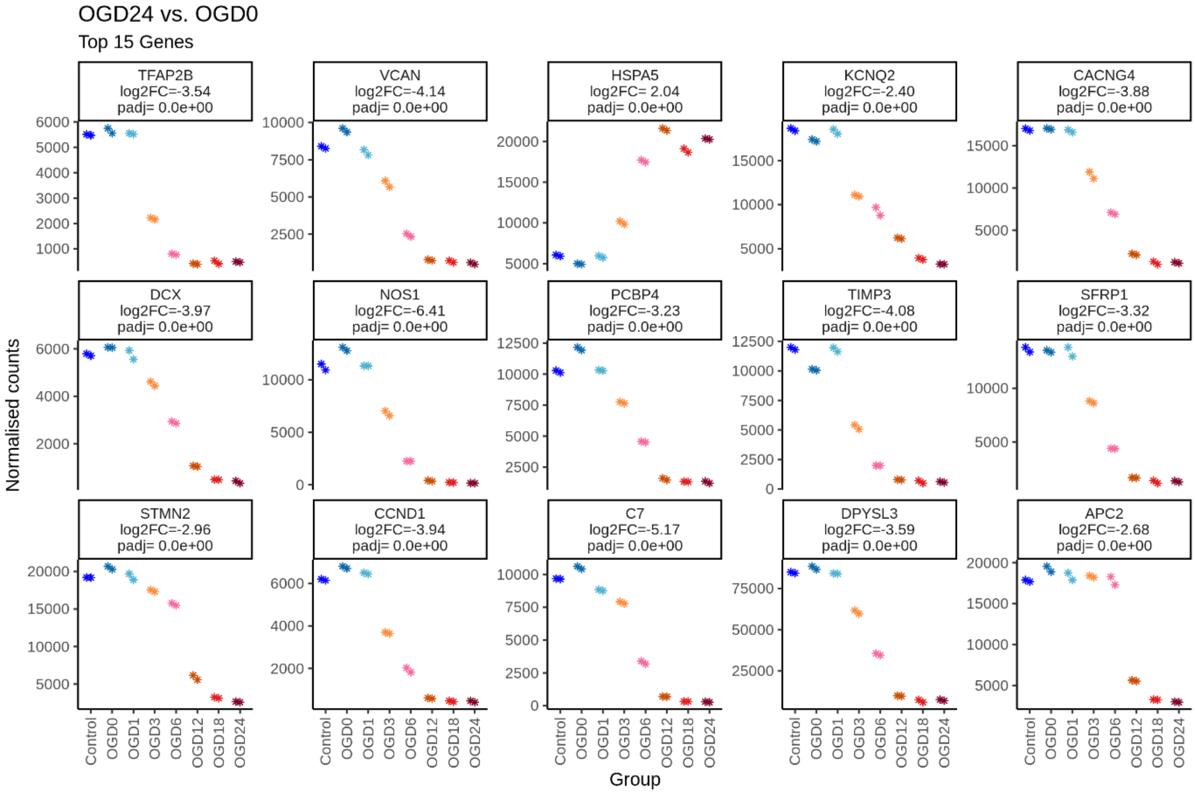

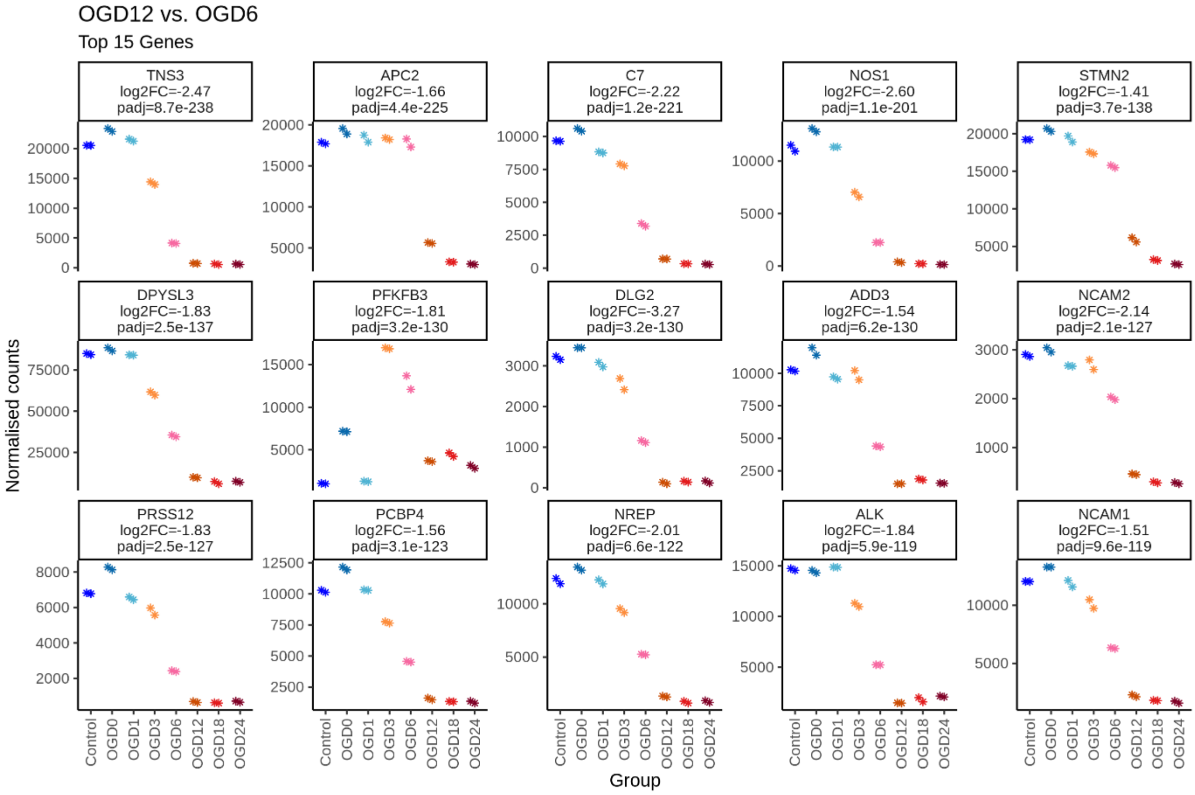

Figure continues to the next page

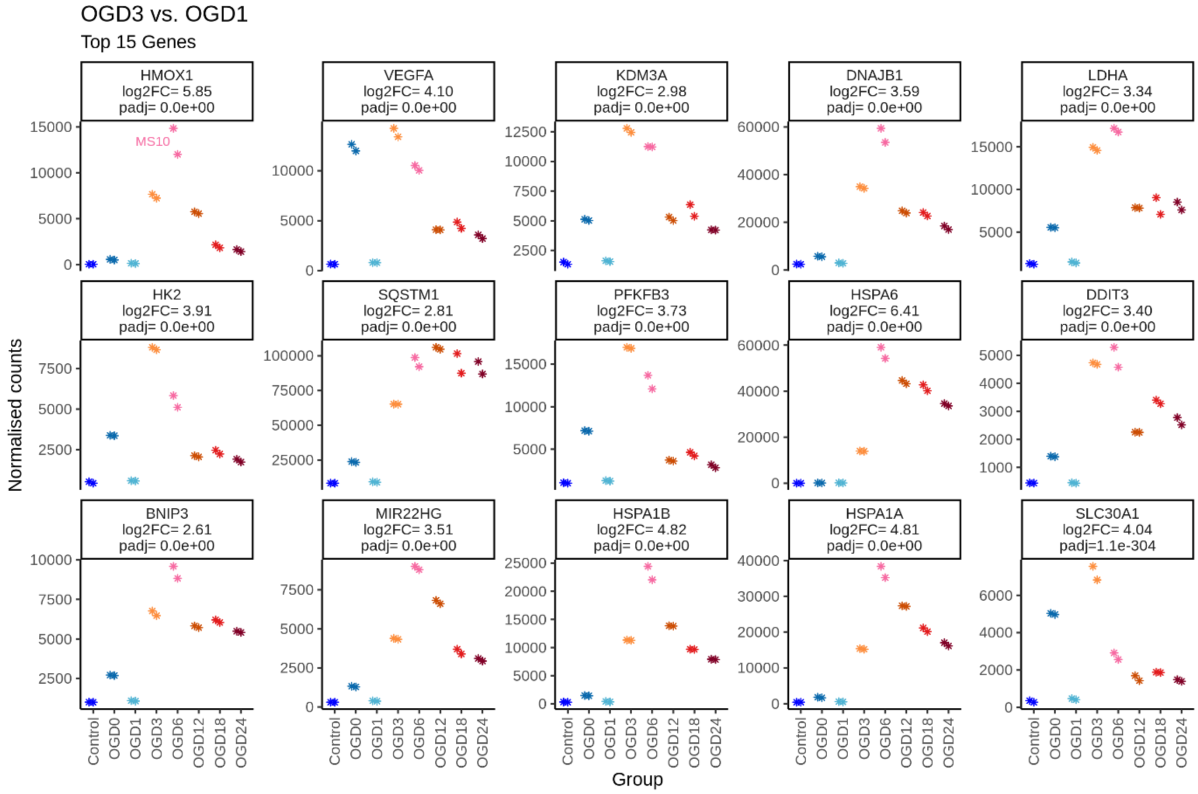

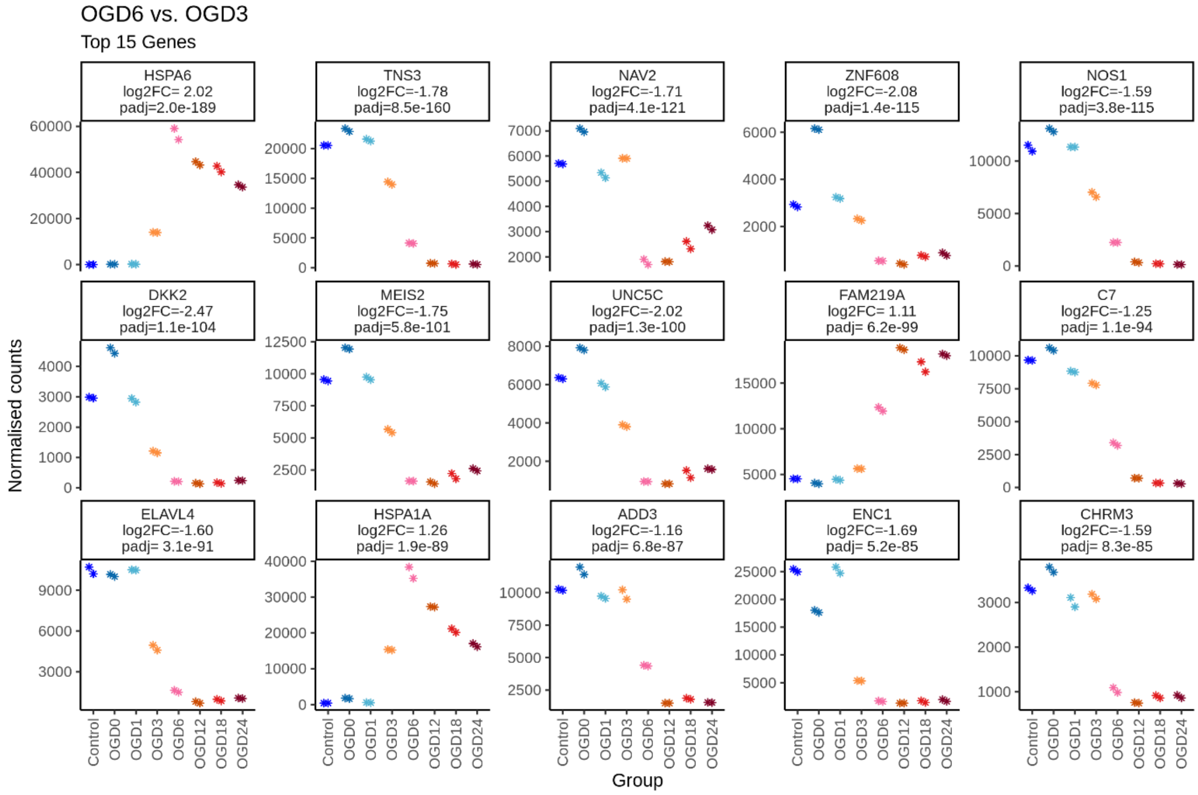

Figure continues to the next page

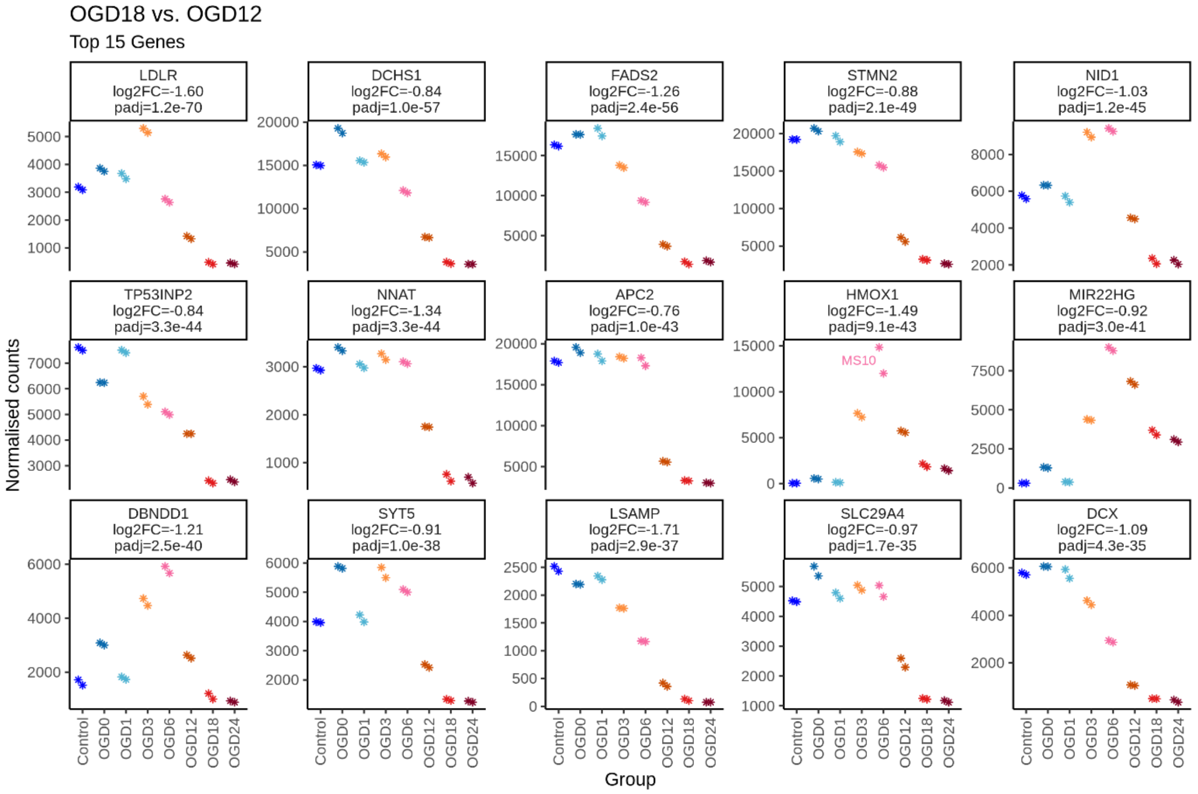

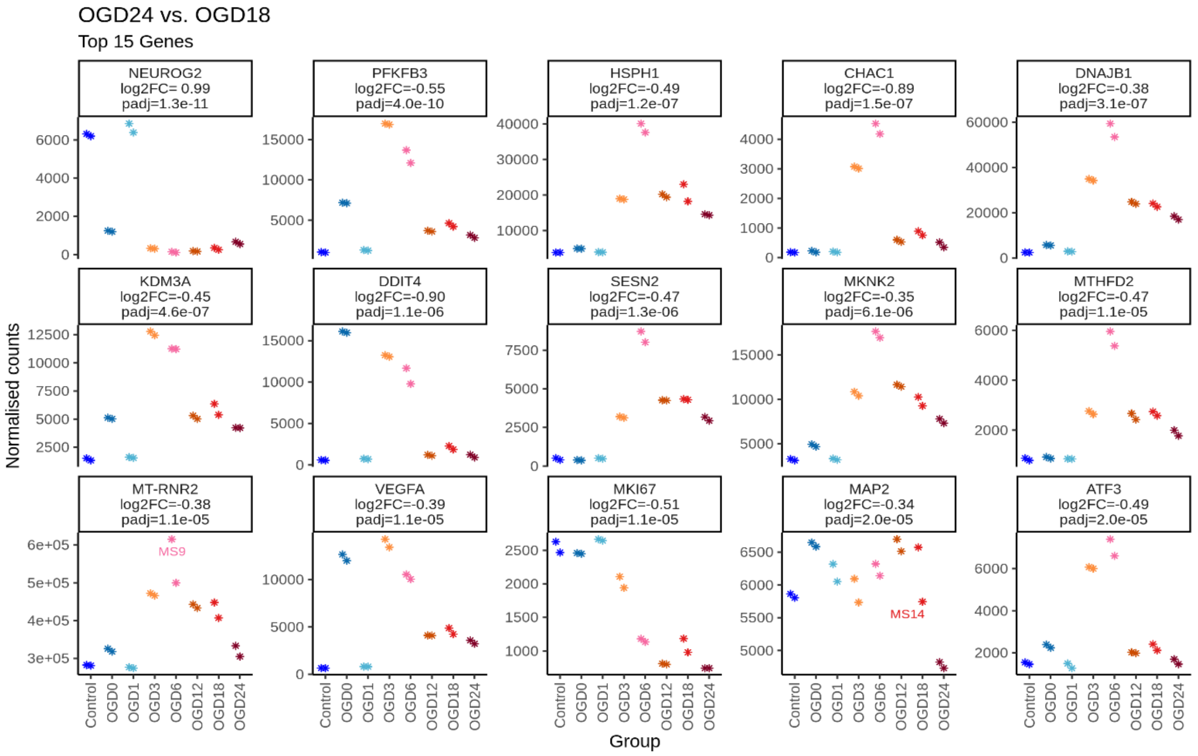

Supplementary Figure S3: Principal Component Analysis (PCA) Based on Differentially Expressed Genes (DEGs).

PCA performed on DEGs across experimental conditions demonstrates consistent clustering of biological replicates and clear separation between control and OGD-treated samples. The observed clustering pattern is consistent with that obtained from whole-transcriptome analysis, supporting the robustness of the dataset.

OGD/R1

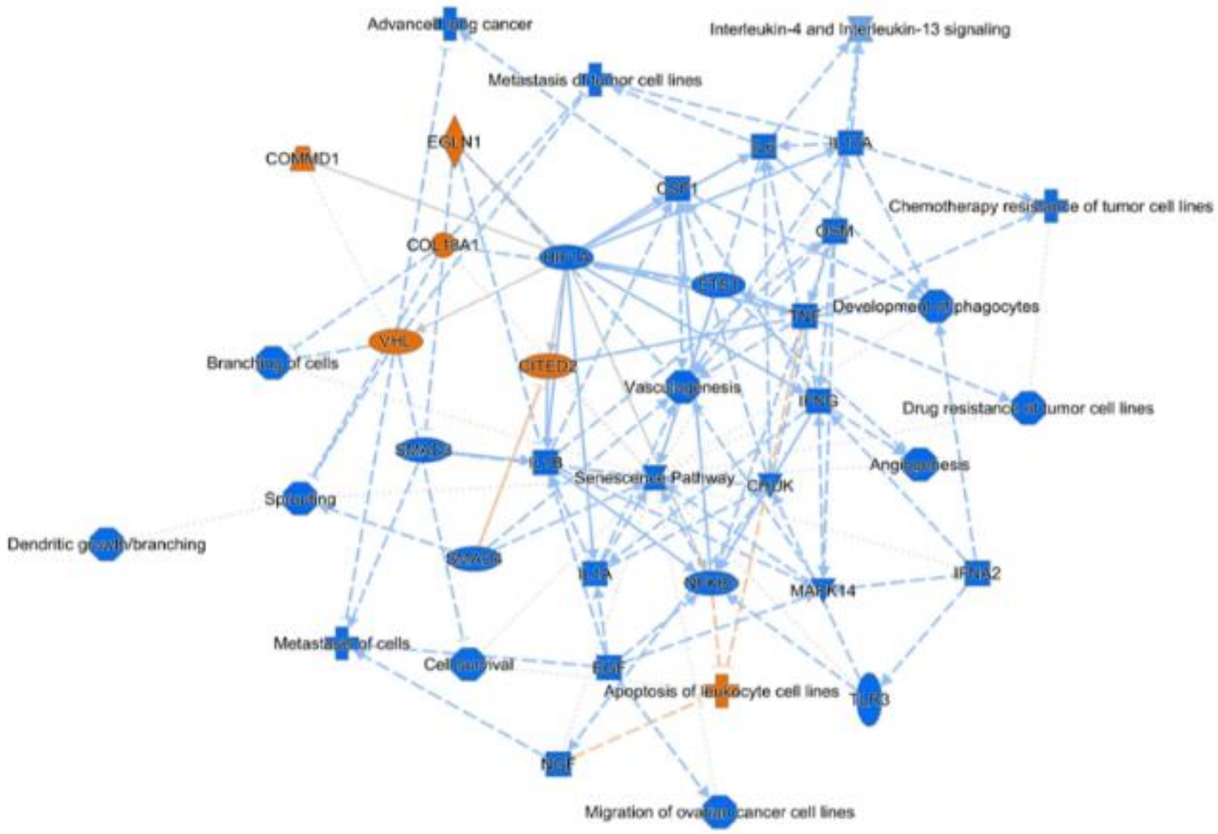

OGD/R3

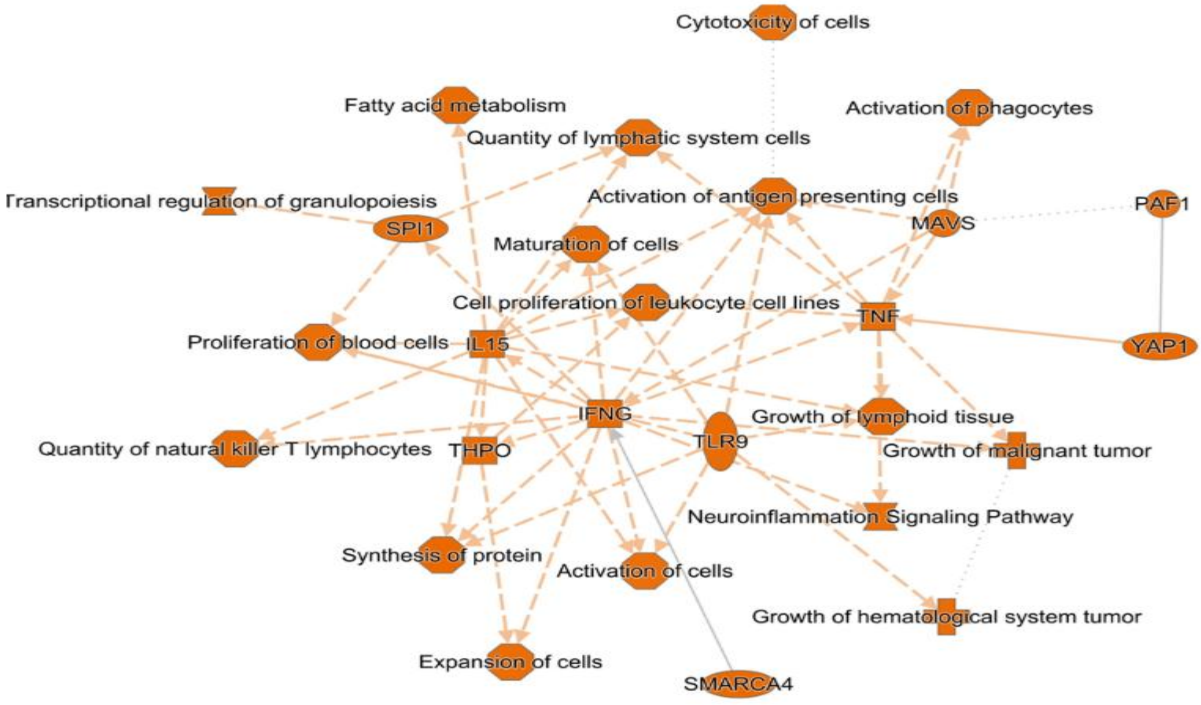

Figure continues to the next page

OGD/R12

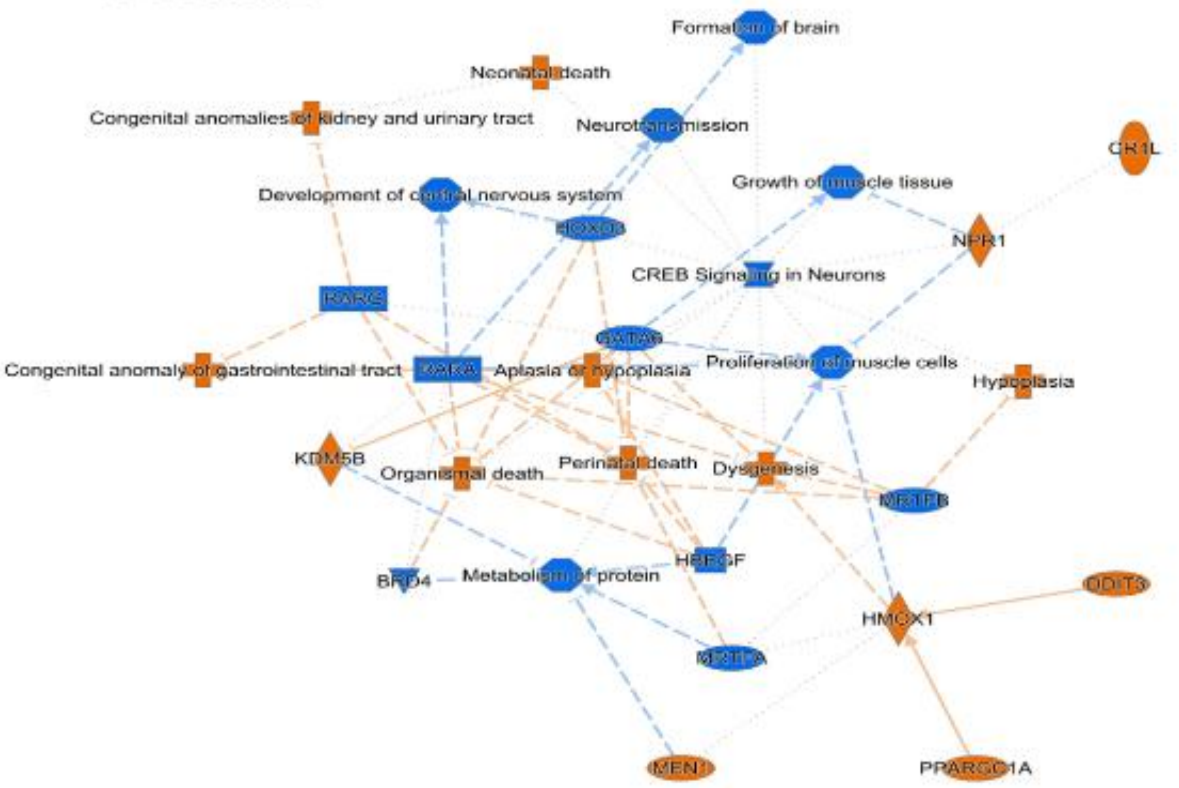

OGD/R18

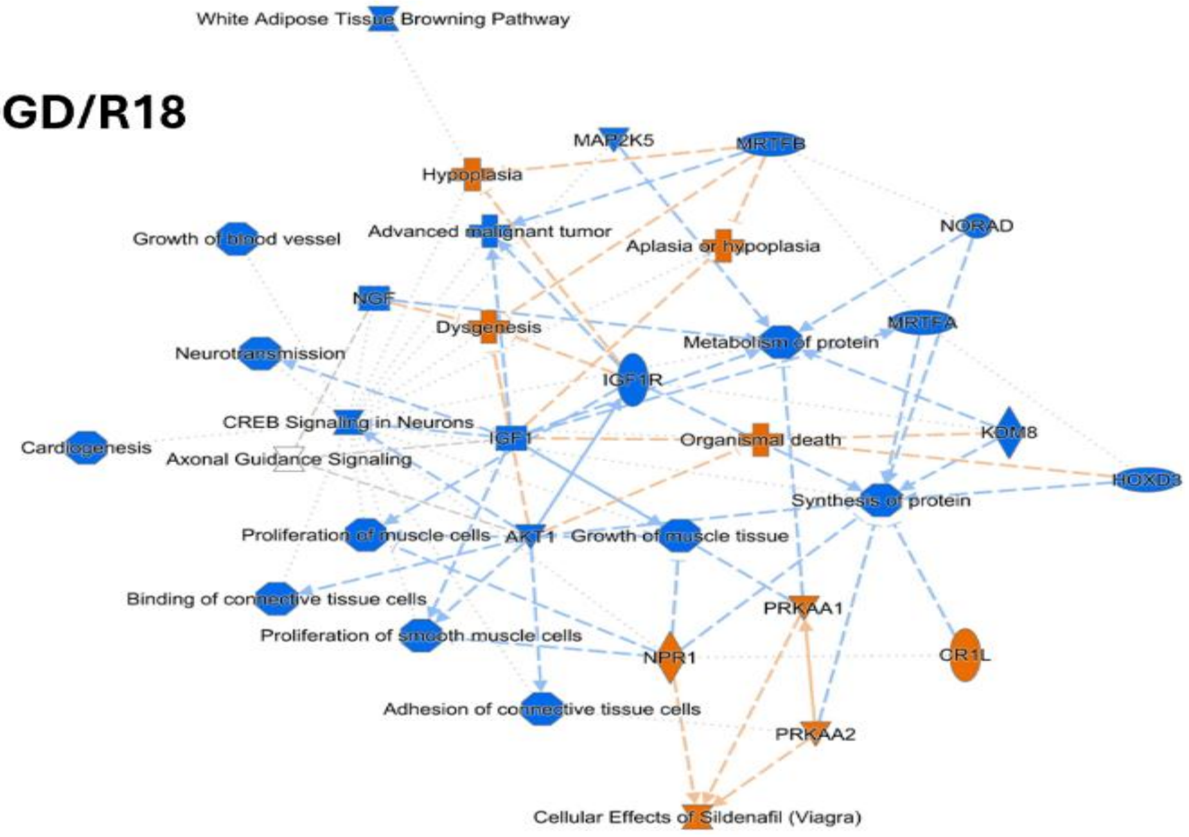

Supplementary Figure S4: Network Analysis Reveals a Transition from Acute Inflammatory Signalling to Late-stage Regenerative Programs in OGD/R-treated Neurons.

IPA-generated networks illustrate the functional connectivity between differentially expressed genes (DEGs) and biological pathways during OGD and reoxygenation at 1 h, 3 h, 6 h, 12 h, 18 h, and 24 h. The initial OGD phase is dominated by pro-inflammatory and hypoxia-responsive hubs, including TNF, IL1B, and HIF-1α, which shift toward a transient transcriptional repression at 1 h reoxygenation. Reoxygenation at 3h–6 h is characterized by a surge in immune-like survival signalling, featuring activation of STAT-mediated pathways and Interleukin (IL2, IL15) hubs. The late-stage recovery is marked by a dual signature: the initiation of neuroregenerative programs, such as axonal guidance and synaptogenesis, which paradoxically coincides with the profound inhibition of the CREB Signalling master switch by 24 h. Key: Nodes represent genes or biological functions, with lines indicating documented biological interactions. Node and edge colours represent the predicted activation state based on experimental log2 fold change: orange indicates predicted activation (z-score > 0), while blue indicates predicted

inhibition (z-score < 0). HIF = Hypoxia-inducible factor.

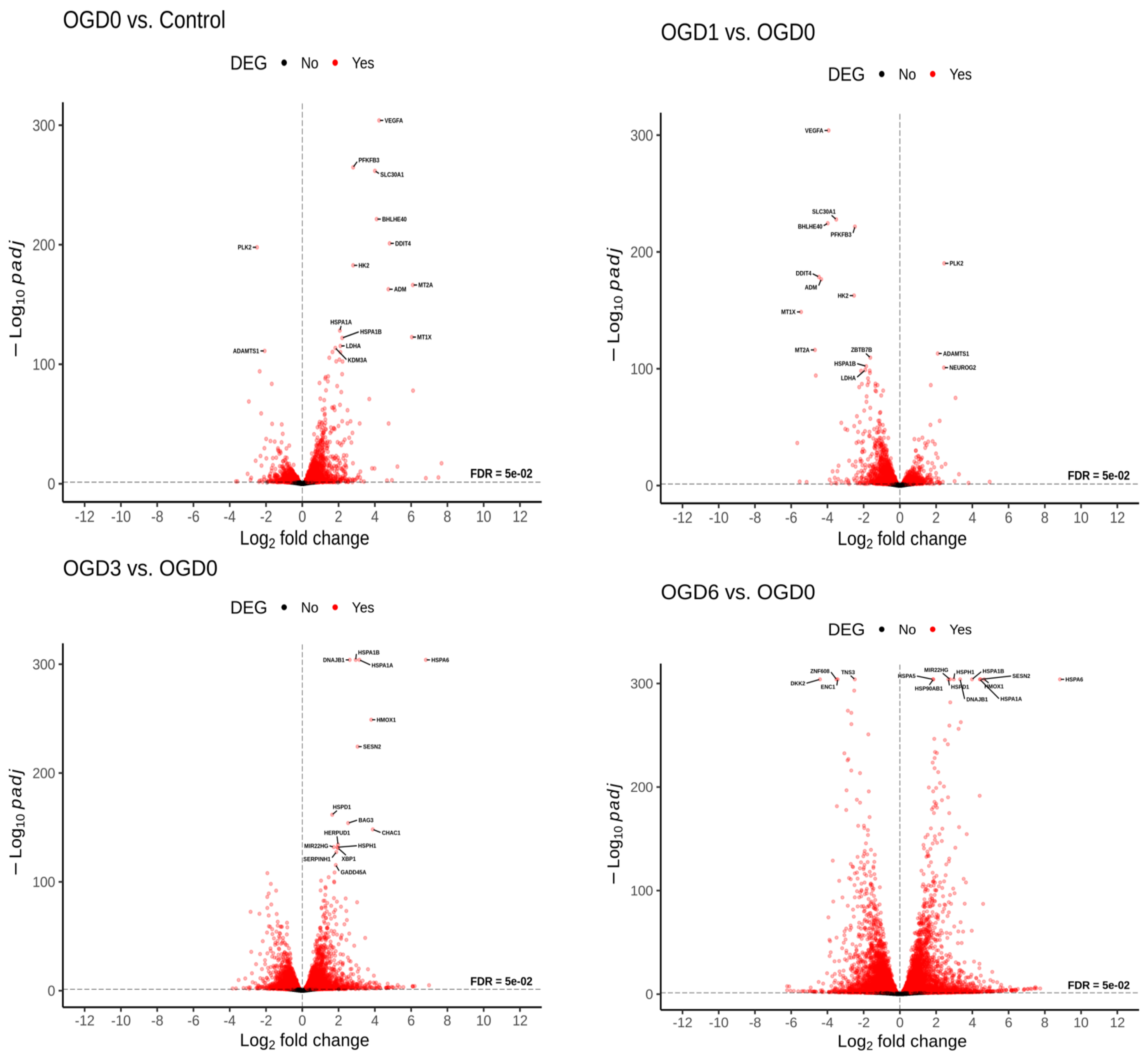

Figure continues to the next page

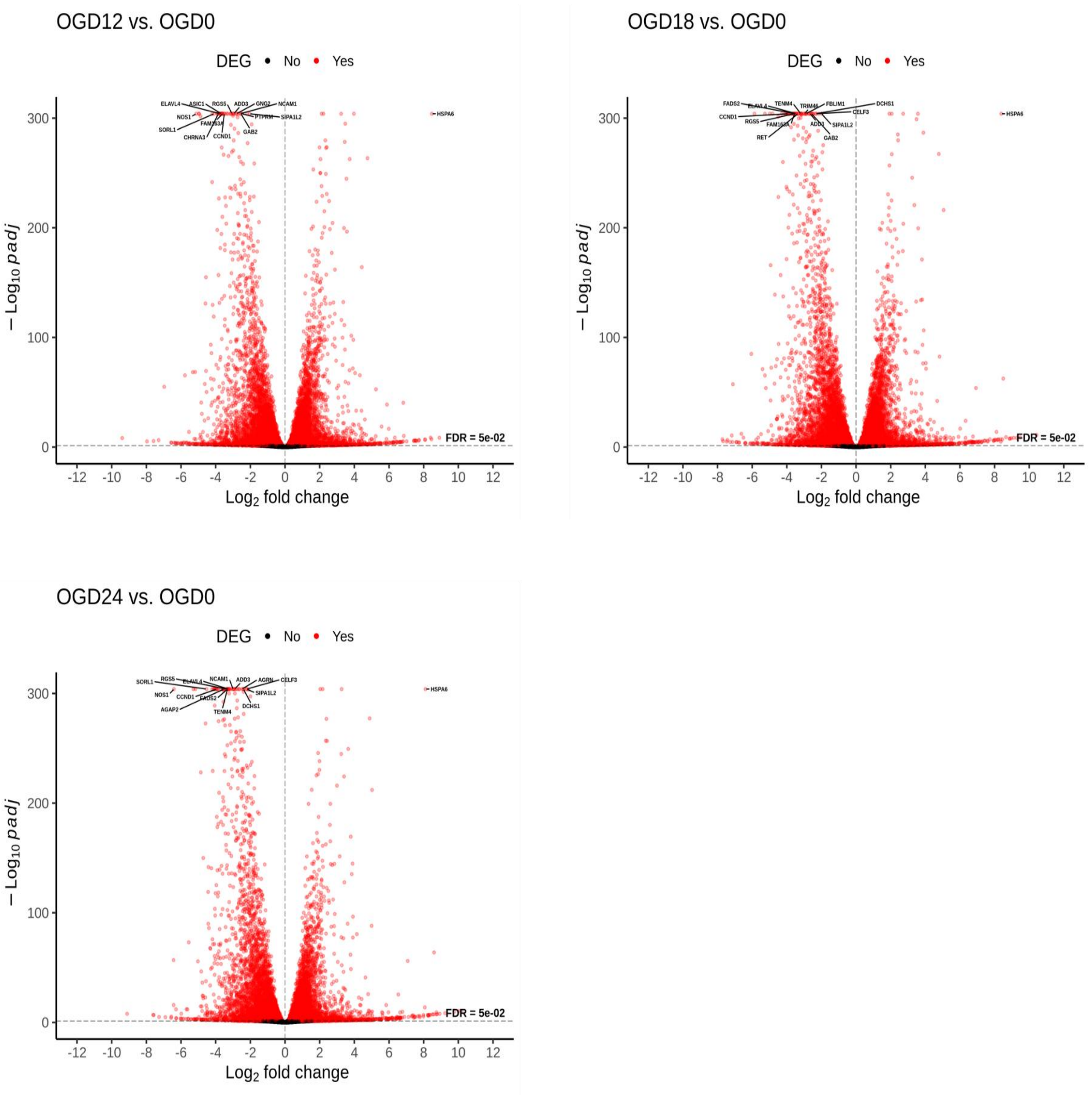

Supplementary Figure S5: Dynamic Transcriptional Profiling of NLCs During OGD and Progressive Reoxygenation.

Volcano plots illustrating the distribution of DEGs across all experimental conditions. The x-axis represents the log2 fold change (LFC), and the y-axis represents the statistical significance (log10 adjusted p-value). Red points indicate genes meeting the significance threshold ($FDR < 0.05). (a) Comparison of OGD vs. Control baseline. (b–g) Comparison of reoxygenation time points (1 h, 3 h, 6 h, 12 h, 18 h, and 24 h) relative to the OGD0 state. Representative top-regulated genes, including hypoxia-responsive elements (VEGFA), heat shock proteins (HSPA6), and neuronal markers (NOS1), are annotated. Key: DEGs = differentially ex-pressed genes, OGD = Oxygen-glucose deprivation, VEGFA = Vascular endothelial growth factor A, HSPA6 = Heat shock protein family A member 6; NOS = Nitric oxide synthase 1.
